# Advances in the Asymmetric Total Synthesis of Natural Products Using Chiral Secondary Amine Catalyzed Reactions of α,β-Unsaturated Aldehydes

**DOI:** 10.3390/molecules24183412

**Published:** 2019-09-19

**Authors:** Zhonglei Wang

**Affiliations:** 1 School of Pharmaceutical Sciences, Tsinghua University, Beijing 100084, China; wangzl16@tsinghua.org.cn; 2 School of Chemistry and Chemical Engineering, Qufu Normal University, Qufu 273165, China

**Keywords:** bioactive natural products, pharmaceuticals, asymmetric total synthesis, chiral secondary amines, α,β-unsaturated aldehydes

## Abstract

Chirality is one of the most important attributes for its presence in a vast majority of bioactive natural products and pharmaceuticals. Asymmetric organocatalysis methods have emerged as a powerful methodology for the construction of highly enantioenriched structural skeletons of the target molecules. Due to their extensive application of organocatalysis in the total synthesis of bioactive molecules and some of them have been used in the industrial synthesis of drugs have attracted increasing interests from chemists. Among the chiral organocatalysts, chiral secondary amines (MacMillan’s catalyst and Jorgensen’s catalyst) have been especially considered attractive strategies because of their impressive efficiency. Herein, we outline advances in the asymmetric total synthesis of natural products and relevant drugs by using the strategy of chiral secondary amine catalyzed reactions of α,β-unsaturated aldehydes in the last eighteen years.

## 1. Introduction

**Scheme 1:**
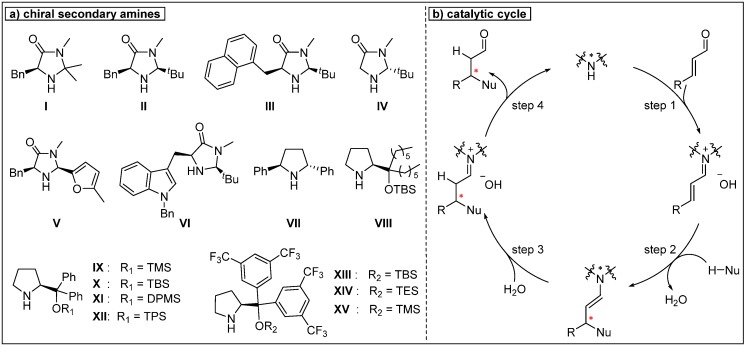
Chiral secondary amines and iminium catalytic cycle.

In 2000, the MacMillan group [1] first described the fundamental concept of LUMO-lowering iminium-ion activation. In 2005, the Jørgensen group [2,3] reported diarylprolinol silyl ether catalysts, which have also been used in iminium-ion catalysis. Since then, chiral secondary amines catalysts (shown in Scheme 1a, including both MacMillan’s catalyst and Jorgensen’s catalyst, not including proline) have been intensively investigated by many groups, becoming one of the most successful areas of organic chemistry [4] and medicinal chemistry [5].

α,β-Unsaturated aldehydes are ubiquitous substrates in the area of asymmetric synthesis. The related mechanistic pathway of catalytic cycle [6,7] for iminium-ion activation is shown in Scheme 1b. The catalytic cycle consists of four principle steps: the first step is formation of the active iminium ion; the second step is conjugate addition reaction (or cycloaddition reaction); the next step is imine formation reaction; the last step is hydrolysis of iminium ion. Of note, the high enantioselectivity was obtained via nucleophiles added to the unshielded *Re*-face of the optically active *E*-configured iminium ion intermediate formed by the chiral secondary amine catalyst and the α,β-unsaturated aldehydes (steric hindrance plays an important role in directing the incoming nucleophile).

Over the past eighteen years, iminium ion activation strategy has extended into the synthesis of complex molecules including natural products and pharmaceuticals. Given the importance of the chiral secondary amines catalysts it is no doubt there have been a lot of reviews on organocatalysis [8,9,10,11,12,13,14]. But the relatively complete review on the total synthesis of natural products and relevant drugs published from 2002 to 2018 by using the strategy of chiral secondary amines catalyzed reactions of α,β-unsaturated aldehydes is still necessary. The present review will focus on the applications of relatively LUMO-lowering catalysis strategy in total synthesis (not including formal synthesis) of natural products.

## 2. Asymmetric Total Synthesis of Bioactive Natural Products and Pharmaceuticals

### 2.1. Friedel-Crafts Alkylation Reaction

The organocatalytic asymmetric Friedel-Crafts alkylation reaction has without question received extensive attention and has evolved to become an important tool in the area of asymmetric total synthesis [15]. As a burgeoning powerful tool, MacMillan’s catalyst is being widely exploited in several important enantioselective reactions, especially in Friedel-Crafts alkylation reactions. The application of chiral secondary amine catalyst in Friedel-Crafts reactions usually requires an electron-rich aromatic compound. It might be pointed out that an acidic co-catalyst may also have an important influence on the enantioselectivity of the asymmetric Friedel-Crafts alkylation reaction.

**Scheme 2:**
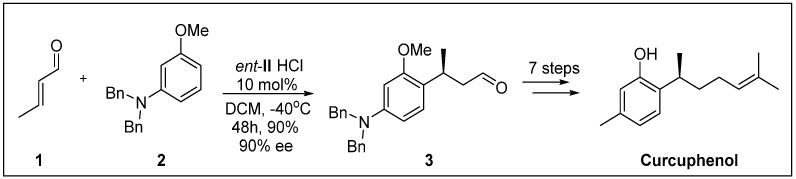
Total synthesis (+)-curcuphenol.

Organocatalytic Friedel-Crafts reactions offer an extraordinarily direct strategy for the straightforward single-step generation of the benzylic chiral center. In 2005, the Kim group [16] accomplished an enantioselective total synthesis of sesquiterpene phenol (+)-curcuphenol (Scheme 2), which was isolated from *Didiscus flavus* [17] and exhibited powerful antifungal activity [18]. The Kim group introduced the stereocenter by a highly enantioselective Friedel-Crafts alkylation reaction.

The key sequence highlighted the use of MacMillan’s catalyst *ent*-II in the presence of crotonaldehyde (**1**), *N*,*N*-dibenzyl-3-anisidine (**2**) and DCM to achieve aldehyde **3** with excellent enantioselectivity (90% *ee*). Further transformations afforded (+)-curcuphenol by a seven-step sequence including Sandmeyer-type bromination and Negishi-type coupling.

**Scheme 3:**
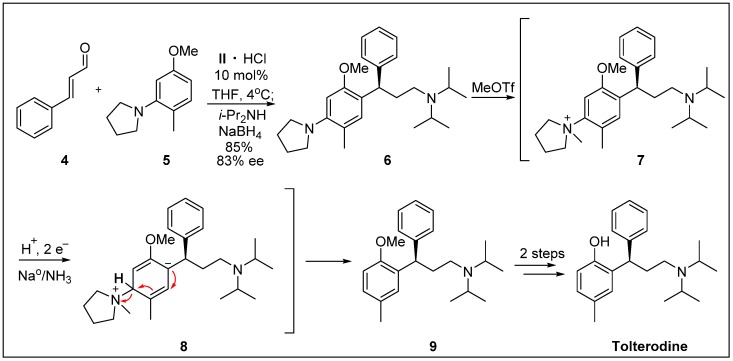
Total synthesis of (+)-tolterodine.

Another impressive example showing the versatility of this methodology was reported in 2009 by the MacMillan group [19] in the excellent total synthesis of bladder-selective antimuscarinic agent (+)-tolterodine (Detrol^®^, Scheme 3). Tolterodine is the first muscarinic receptor antagonist developed to treat overactive bladder [20]. The group employed an enantioselective organocatalytic alkylation of aniline with α,β-unsaturated aldehyde developed by the same group in 2002.

This key sequence involved the use of MacMillan’s catalyst II in the presence of α,β-unsaturated aldehyde **4**, aniline **5**, and THF to achieve β-branched aldehyde in 88% yield with 83% ee via a Friedel-Crafts alkylation. Remarkably, a reductive amination reaction rapidly occurred upon treating the β-branched aldehyde with *i*-Pr_2_NH using NaBH_4_ as a reducing agent to afford tertiary amine **6** in 97% yield. Further transformations afforded the pharmaceutical agent (+)-tolterodine by a three-step sequence including an aryl ammonium reduction (Na^0^/NH_3_) devised by the same group.

**Scheme 4:**
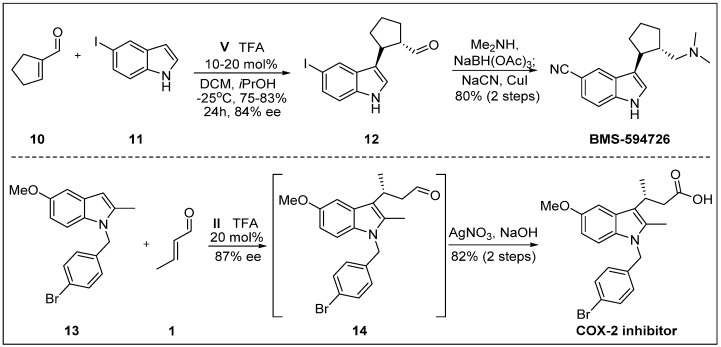
Total synthesis of (+)-BMS-594726 and COX-2 inhibitor.

Indoles represent another important type of nucleophiles that has been widely used in Friedel-Crafts reactions. In 2005, Bristol Myers Squibb [21] developed an excellent 3-step total synthesis of selective serotonin reuptake inhibitor (+)-BMS-594726 (Scheme 4), which is the first example of α-branched α,β-unsaturated aldehyde being used as electrophile in an organocatalytic Friedel-Crafts alkylation.

This key sequence highlighted the use of MacMillan’s catalyst V in the presence of α-branched α,β-unsaturated aldehyde **10**, 5-iodoindole (**11**), *i*PrOH and DCM to obtain aldehyde **12** with 84% *ee*. Further transformations afforded (+)-BMS-594726 on 11.3 g scale by a simple two-step sequence including reductive amination and subsequent Buchwald cyanation. In addition, the MacMillan group [22] reported an excellent two-step total synthesis of COX-2 inhibitor (Scheme 4) in 87% *ee* via their own developed organocatalytic Friedel-Crafts indole alkylation protocol.

**Scheme 5:**
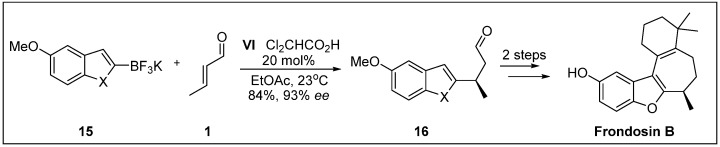
Total synthesis of (+)-frondosin B.

In 2010, the MacMillan group [23] reported another excellent three-step total synthesis of sesquiterpene (+)-frondosin B (Scheme 5), which had been first isolated from *Dysidea frondosa* in 1997 [24] and exhibited potential anti-HIV activity [25]. The group employed a novel organocatalytic Michael addition for the construction of its stereogenic center.

This key sequence highlighted the use of MacMillan’s catalyst in the presence of α,β-unsaturated aldehyde **1**, activated boronate species **15 [26]**, dichloroacetic acid and EtOAc to achieve aldehyde in 84% yield with excellent enantioselectivity (93% *ee*). Final transformation from to (+)-frondosin B was achieved by a very efficient two-step procedure including allylic alkylation, allylic Friedel-Crafts alkylation, olefin isomerization, and demethylation (the last three transformations counted as one step). This novel organocatalytic approach culminated in the shortest asymmetric total synthesis of (+)-frondosin B to date [27]. This impressive example that perfectly illustrates the power of Friedel-Crafts reaction in the asymmetric total synthesis of natural products.

**Scheme 6:**
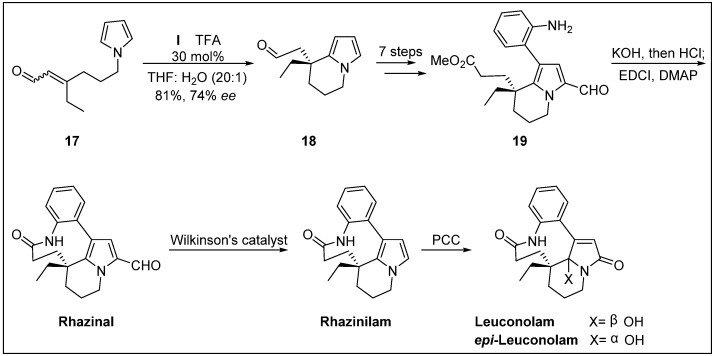
Total synthesis of (−)-rhazinal, (−)-rhazinilam, and (−)-leuconolam.

In 2006, the Banwell group [28] accomplished the enantioselective total synthesis of four alkaloids (−)-rhazinal, (−)-rhazinilam, (−)-leuconolam, and (+)-*epi*-leuconolam with reasonable enantioselectivity (Scheme 6) via organocatalytic intramolecular Friedel-Crafts reactions.

Although the above interesting targets are structurally complex, the key precursor aldehyde **18** only contains one stereocenter, which can be introduced by carrying out a chiral secondary amine I-catalyzed Friedel-Crafts alkylation of the starting material **17**.

**Scheme 7:**
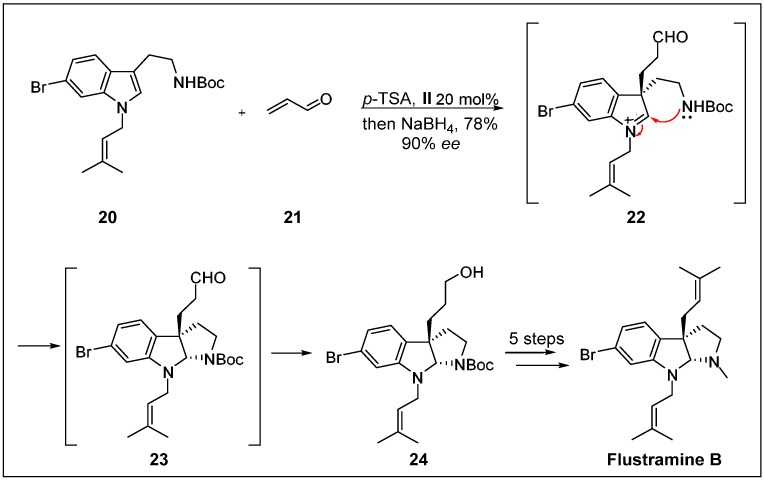
Total synthesis of (-)-flustramine B.

In 2004, the MacMillan group [29] accomplished the first enantioselective total synthesis of the pyrroloindoline alkaloid (−)-flustramine B (Scheme 7), which was isolated from the bryozoan *Flusta foliacea* [30]. This work provides a rapid and straightforward methodology for the stereoselective construction of its quaternary carbon stereocenters for the first time (indeed, before is work, only two racemic syntheses of flustramine B had been reported).

The key strategy in this elegant total synthesis was a highly enantioselective cascade Friedel-Crafts/cyclization reaction. This sequence highlighted the use of MacMillan’s catalyst II in the presence of α,β-unsaturated aldehyde **21**, and 6-bromotryptamine derivative **20** to obtain key tricyclic intermediate **23**, which was reduced using NaBH_4_ to achieve key tricyclic intermediate **24** in 78% yield with excellent enantioselectivity (90% *ee*). After five additional steps, the total synthesis of (−)-flustramine B was accomplished. The key sequence included a H_2_O_2_-mediated selenoxide elimination, Grubbs olefin metathesis reaction and reduction. It is worth mentioning that the above case is the first application of organocatalytic cascade strategy in synthesis of natural products. 

**Scheme 8:**
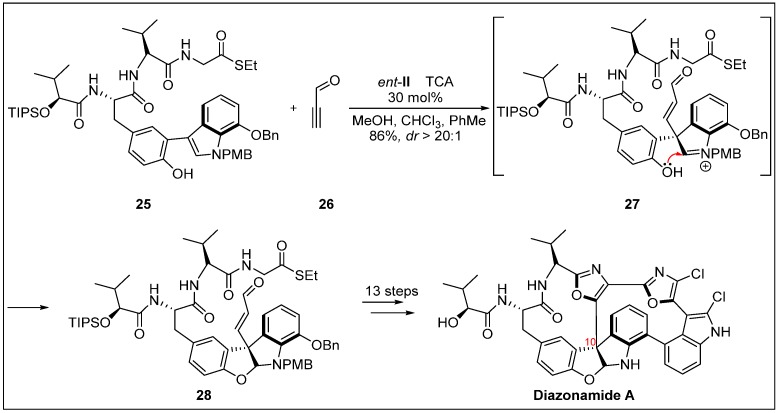
Total synthesis of (−)-diazonamide A.

In 2011, the MacMillan group [31] developed an excellent 20-step total synthesis of the 12-membered macrocycle (−)-diazonamide A (Scheme 8), which was isolated initially from the ascidian *Diazona angulata*. in 1991 by the Fenical group [32] and exhibited powerful antimitotic activity [33]. The key strategy in this elegant total synthesis was a highly enantioselective cascade Friedel-Crafts/cyclization reaction.

The unique triaryl-substituted quaternary stereocenter at C-10 makes this compound an especially challenging target for synthesis. The key sequence highlighted the use of MacMillan’s catalyst in the presence of alkynal **26**, indole derivative **25**, TCA, MeOH, CHCl_3_, and PhMe to achieve tricyclic intermediate **28** in 86% yield with high stereoselectivity (>20:1 *dr*) via intermolecular Friedel-Crafts reaction, followed by an intramolecular nucleophilic addition of **27**. Further transformations afforded (−)-diazonamide A by a thirteen-step sequence, making it another impressive example of organocatalytic cascade strategy.

### 2.2. Michael Addition Reaction

Organocatalytic asymmetric Michael addition reaction is one of the most general, convenient, powerful, and versatile tools for the formation of C-C bonds, C-N bonds, C-O bonds and so on [34]. It is interesting to note that Jorgensen’s catalyst has received much more extensive in the field of both asymmetric intramolecular and intermolecular Michael addition reactions.

#### 2.2.1. Oxa-Michael Addition

**Scheme 9:**
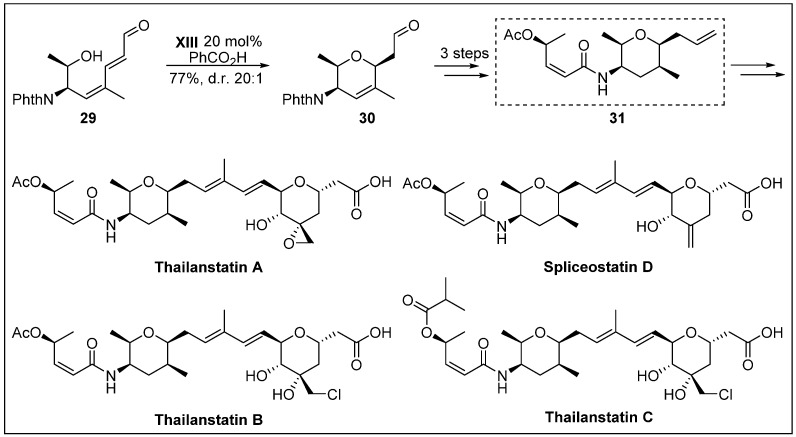
Total synthesis of thailanstatins A-C and spliceostatin D.

Chiral secondary amine catalysts paved the way for an effective method for the asymmetric synthesis of valuable tetrahydropyran rings with excellent stereoselectivity. In 2016, the Nicolaou group [35] accomplished a short and efficient first total synthesis of spliceosome inhibitor thailanstatin A (Scheme 9), which had been first isolated from *Thailandensis burkholderia* MSMB43 in 2013 by the Cheng group [36] and exhibited significant cancer activity. The key reaction in this elegant total synthesis was a highly enantioselective intramolecular *oxa*-Michael addition.

After several preliminary steps, the synthetic precursor **29** of the key organocatalytic reaction could be prepared from the known Garner aldehyde starting material. Thus, when α,β,γ,δ-unsaturated aldehyde **29** was treated with catalyst XIII as well as benzoic acid in the presence of DCM at 0 °C, the intramolecular *oxa*-Michael addition proceeded in a highly enantioselective manner to produce tetrahydropyran ring **30** in 77% yield (dr > 20:1). Then advanced intermediate **31** was prepared through a sequence including stereoselective hydrogenation, methylenation and an amide formation reaction. Final transformation from amide **31** to thailanstatin A was achieved by a two-step sequence including cross metathesis and Suzuki cross-coupling. Further, thailanstatin B, thailanstatin C, spliceostatin D, and an array of analogues (Scheme 9) have been prepared by the Nicolaou group [37] in 2018 via the key organocatalytic intramolecular 1,4-addition strategy.

**Scheme 10:**
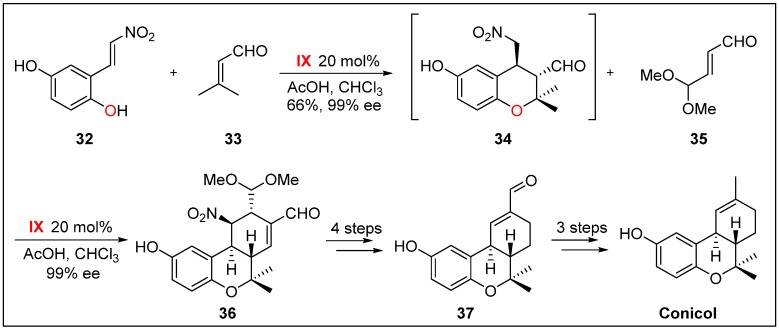
Total synthesis of (+)-conicol.

Organocatalytic three-component cascade reaction provides a straightforward entry to target molecule bearing three contiguous stereocenters. In 2010, the Hong group [38] described the first asymmetric total synthesis of (+)-conicol (Scheme 10), which had been first isolated from ascidian *Aplidium conicum* in 2002 by the Salvá group [39]. The key reaction in this elegant synthesis was a highly enantioselective three-component one-pot quadruple domino reaction, namely, *oxa*-Michael addition/Michael addition/Michael addition/aldol condensation.

This key sequence highlighted the use of Jorgensen’s catalyst IX in the presence of α,β-unsaturated aldehydes **33** and **35**, (*E*)-2-(2-nitrovinyl)-benzene-1,4-diol (**32**), acetic acid, and CHCl_3_ to provide hexahydro-6*H*-benzo[*c*]chromene **36** with superb enantioselectivity (99% *ee*) via a domino *oxa*-Michael addition/Michael addition sequence of **32**, followed by a domino nitro-Michael addition/aldol condensation of **34**. Further transformations afforded (+)-conicol by a seven-step sequence including RhCl(PPh_3_)_3_ decarbonylation, hydrogenation, and metallic reduction.

**Scheme 11:**
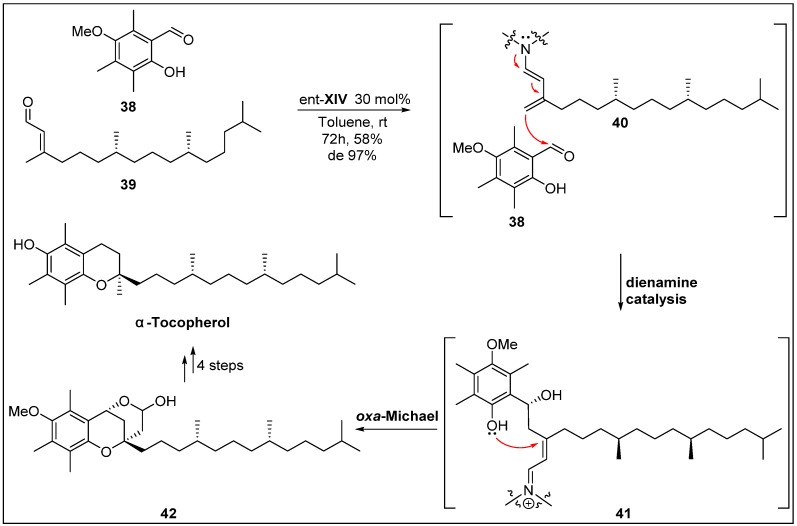
Total synthesis of α-tocopherol.

α-Tocopherol is one of the most biologically significant members of the vitamin E family. In 2008, the Woggon group [40] published an high diastereoselective three-component cascade γ-functionalization/*oxa*-Michael addition reaction using chiral secondary amines catalyst *ent*-XIV (Scheme 11).

Mechanistically, the transformation occurs between aldehyde **38** and **40** via the intermediary formation of phenol ion **41**, which is in turn readily trapped in an intramolecular way by the hydroxy group, giving rise to key intermediate **42**. Then α-tocopherol was obtained successfully by a standard four step transformation.

**Scheme 12:**
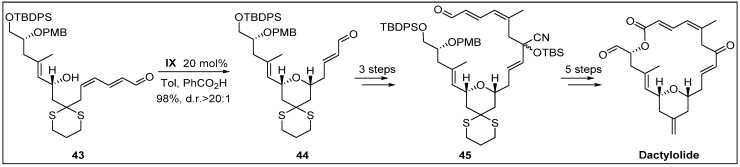
Total synthesis of (+)-dactylolide.

In 2012, the Hong group and the Kim group [41] published the enantioselective total synthesis of 20-membered macrolide (+)-dactylolide (Scheme 12), which had been first isolated by the Riccio group in 2001 from a *Dactylospongia* sp. sponge and exhibited modest antitumor activity [42]. The key reaction in this elegant total synthesis was a highly enantioselective intramolecular 1,6-*oxa* Michael addition reaction.

Synthetic precursor **43** of the key conjugate addition reaction was prepared by a seven-step transformation from known 1,3-dithiane-2-ethanol. Then the key sequence highlighted the use of Jorgensen’s catalyst I in the presence of α,β,γ,δ-unsaturated aldehyde **43**, benzoic acid, and toluene to achieve 2,6-*cis*-disubstituted methylene tetrahydropyran enal **44** in 98% yield with excellent stereoselectivity (>20:1 dr) via an organocatalytic *oxa*-Michael addition reaction. Further transformations gave rise to (+)-dactylolide via an eight steps sequence including intramolecular NHC-catalyzed oxidative macrolactonization reaction, Wittig olefination, and Dess-Martin oxidation.

#### 2.2.2. Aza-Michael Addition

The Michael addition which uses a nitrogen negative-ion as donor to form the C-N bond, is called the *aza*-Michael addition. α-Substituted nitrogen-containing heterocycles bearing a stereocenter are another kind of natural products that can be converted efficiently using chiral secondary amines catalysts.

**Scheme 13:**
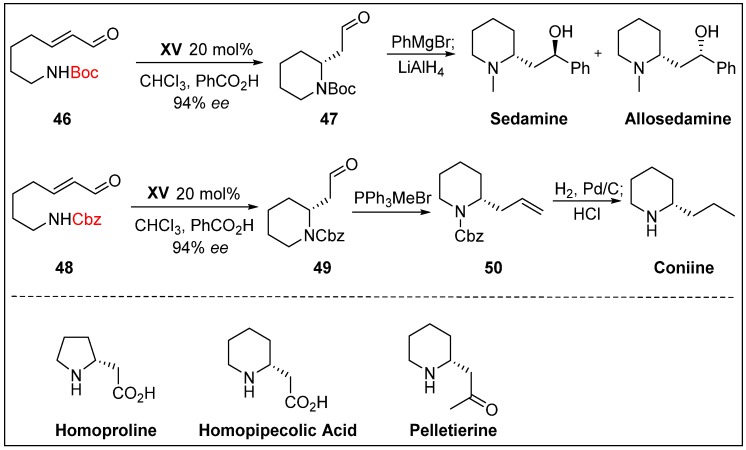
Total synthesis of (+)-sedamine, (+)-allosedamine, (+)-coniine, (−)-homoproline, (−)-homopipecolic acid, and (−)-pelletierine.

In 2007, the Fustero group [43] accomplished the enantioselective total syntheses of three piperidine alkaloids (Scheme 13), namely, (+)-allosedamine, (+)-sedamine, and (+)-coniine starting from similar precursors **47** and **49**. Synthetic precursors were prepared via an organocatalytic asymmetric intramolecular *aza*-Michael reaction.

This asymmetric cyclisation reaction highlighted the use of Jorgensen’s catalyst XV in the presence of α,β-unsaturated aldehyde **46** (or **48**), benzoic acid and CHCl_3_ to achieve piperidine aldehyde **47** (or **49**) in excellent enantioselectivity (94% *ee*). Further transformations afforded the above three alkaloids by a simple two to four steps sequence.

**Scheme 14:**
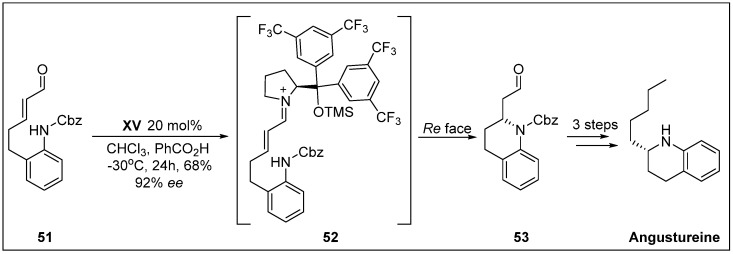
Total synthesis of (+)-angustureine.

In 2008, the Carter group [44] accomplished the enantioselective total syntheses of three other alkaloids, namely, (−)-homoproline, (−)-homopipecolic acid, and (−)-pelletierine using Jorgensen’s catalyst XV via the same organocatalytic asymmetric intramolecular *aza*-Michael reaction (Scheme 13). The same year, the Fustero group [45] also described the total synthesis of tetrahydroquinoline alkaloid (+)-angustureine (Scheme 14) using the above organocatalytic *aza*-Michael strategy.

**Scheme 15:**
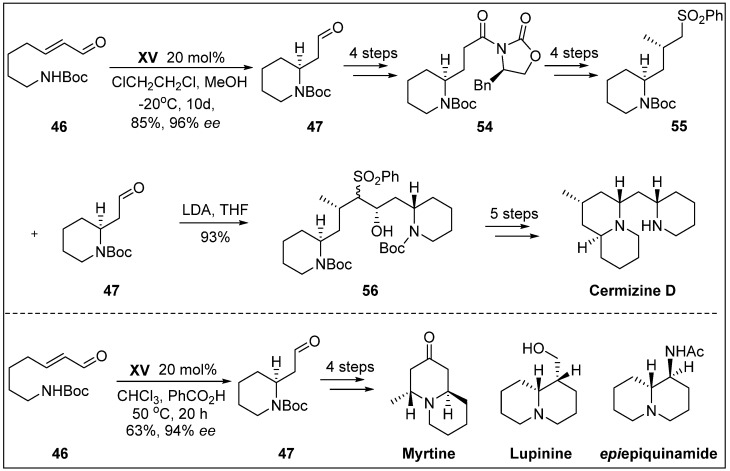
Total synthesis of (+)-cermizine D, (+)-myrtine, and (−)-lupinine.

In 2012, the Carter group [46] accomplished the elegant total synthesis of piperidine alkaloid (+)-cermizine D (Scheme 15), which had been isolated in 2004 by the Kobayashi group [47] from the Chinese herbal medicine *Lycopodium cernuum* and exhibited modest cytotoxicity. The key reaction in this efficient synthesis was an enantioselective intramolecular *aza*-Michael reaction using almost the same protocol as the Fustero group.

With piperidine **47** in hand, a subsequent eight-step transformation provided another precursor sulfone **55**, which gave hydroxy sulfone **56** via deprotonation of sulfone **55** followed by addition of piperidine aldehyde **47**. Further transformations afforded (+)-cermizine D by a five-step sequence including oxidation, reduction, desulfurization, and S_N_2 cyclization reaction. It is worth note that this short as well as practical total synthesis has proven to be an extremely powerful tool by taking advantage of the common intermediate strategy.

Moreover, the Fustero group [48] accomplished the enantioselective total syntheses of other three quinolizidine alkaloids, namely, (+)-myrtine, (−)-lupinine, and (+)-*epi*epiquinamide via the same asymmetric *aza*-Michael reaction (Scheme 15).

**Scheme 16:**
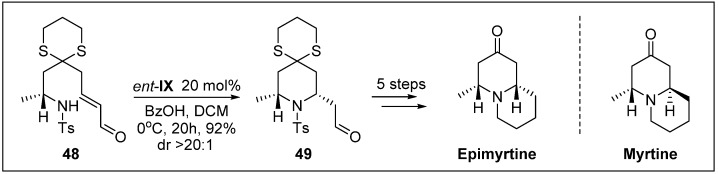
Total synthesis of (−)-epimyrtine and (+)-myrtine.

In 2011, the Hong group [49] reported the total synthesis of quinolizidine alkaloid (−)-epimyrtine (Scheme 16), which had been isolated from *vaccinium myrtillus* in 1981 by the Hootelé group [50] and exhibited potential anticancer activity [51].

The group employed an intramolecular highly stereoselective *aza*-Michael reaction for the construction of its 2,6-*cis*-piperidine block by taking advantage of an iminium activation strategy. In addition, (+)-myrtine has also been prepared from the common substrate.

**Scheme 17:**
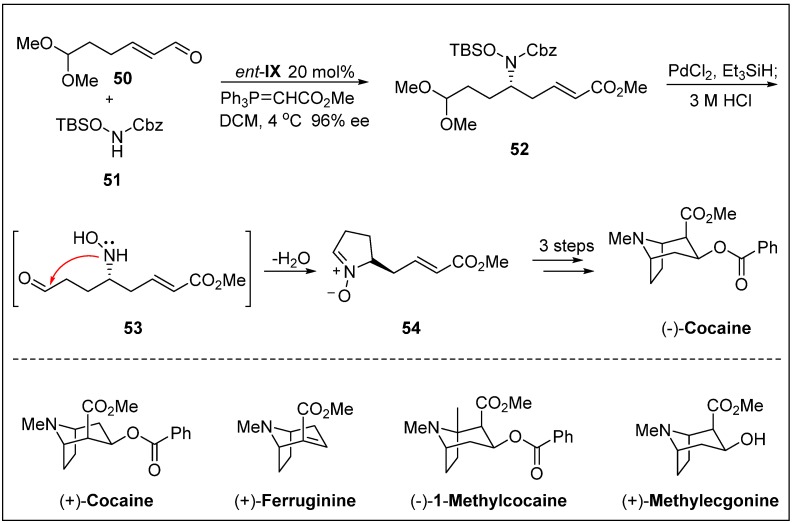
Total synthesis of (−)-cocaine, (+)-cocaine, (+)-ferruginine, (−)-1-methylcocaine, and (+)-methylecgonine.

In 2012, the Córdova group [52] described an excellent total synthesis of the tropane alkaloid (−)-cocaine (Scheme 17), which had been first isolated in 1895 by the Neiman group and exhibits powerful analgesic activity [53]. The group employed a highly enantioselective one-pot *aza*-Michael/Wittig reaction for the construction of its chiral building block.

The key sequence highlighted the use of Jorgensen’s catalyst *ent*-IX in the presence of α,β-unsaturated aldehyde **50**, hydroxylamine **51**, phosphonium ylide and DCM to achieve α,β-unsaturated δ- amino acid **52** with excellent enantioselectivity (96% *ee*). Further transformations afforded (−)-cocaine by a five-step sequence including deprotection, cyclization, intramolecular 1,3-dipolar cycloaddition, hydrogenation, and benzoylation. In addition, the total synthesis of other four structurally related alkaloids, namely, (+)-cocaine, (+)-ferruginine, (−)-1-methylcocaine, and (+)-methylecgonine were prepared via this organocatalytic three-component tandem *aza*-Michael/Wittig reaction (Scheme 17).

#### 2.2.3. Michael Addition

The Michael addition is one of the most important and classical reactions of organic chemistry. Organocatalytic enantioselective Michael addition reactions have been widely used as key steps of all kinds of important natural products and drugs.

**Scheme 18:**
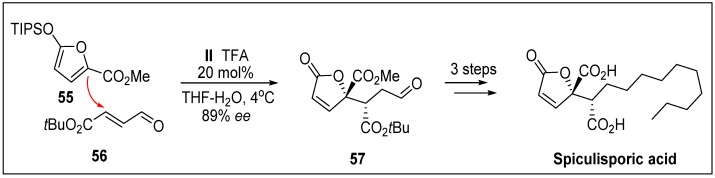
Total synthesis of (−)-spiculisporic acid.

The iminium-catalyzed enantioselective Mukaiyama-Michael addition reaction overcomes the deficiency of Lewis acids enabling 1,4-additions of silyloxy furans to α,β-unsaturated aldehydes. In 2003, the MacMillan group [54] reported an elegant four-step total synthesis of butenolide (−)-spiculisporic acid (Scheme 18), utilizing an unprecedented organocatalytic Mukaiyama-Michael addition reaction.

Mechanistically, Mukaiyama-Michael addition of the silyloxyfuran **55** to the acceptor α,β-unsaturated aldehyde **56** catalyzed by MacMillan’s catalyst II furnished the key intermediate **57** in 90% yield with good enantioselectivity (89% *ee*).

**Scheme 19:**
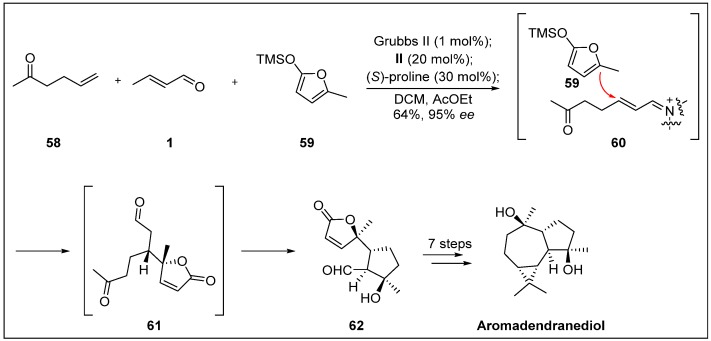
Total synthesis of (−)-aromadendranediol.

In 2009, the MacMillan group [55] reported the elegant total synthesis of tricyclic sesquiterpene diol (−)-aromadendranediol (Scheme 19), which had been first isolated in 1978 from the coral *Sinularia mayi* by the Djerassi group [56]. The key reaction in this novel synthesis was a triple cascade cross-metathesis reaction/Mukaiyama-Michael addition/intramolecular aldol reaction.

Synthetic precursor **60** of the key conjugate addition was prepared by a cross-metathesis reaction of **58** with **1**. Then the key sequence highlighted the use of MacMillan’s catalyst II in the presence of α,β-unsaturated aldehyde **60**, silyloxyfuran **59**, DCM, and EtOAc to achieve keto-aldehyde **61** with 95% *ee*. A subsequent intramolecular aldol addition afforded the advanced precursor **62**. Further transformations gave rise to (−)-aromadendranediol via a seven step sequence including global reduction, chemoselective oxidation, and Wittig olefination.

**Scheme 20:**
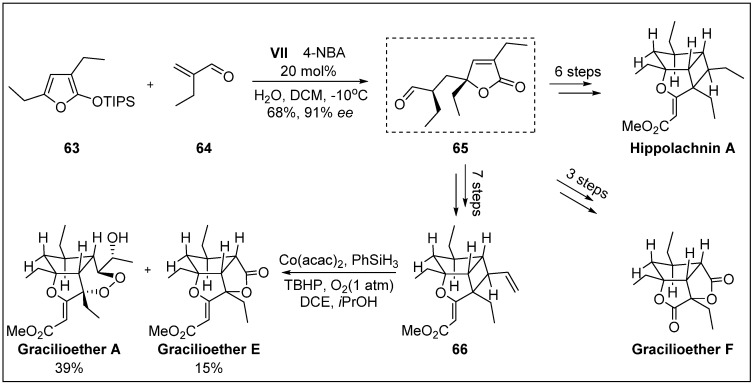
Total synthesis of (+)-hippolachnin A, (+)-gracilioethers A, E and F.

In 2018, the group of Tang [57] accomplished the novel enantioselective total syntheses of four diverse *Plakortin* polyketides [58,59], namely, (+)-hippolachnin A, (+)-gracilioether A, (−)-gracilioether E and (−)-gracilioether F starting from the common precursor **65**, which was afforded via organocatalytic Mukaiyama-Michael reaction (Scheme 20).

The key sequence highlighted the use of diphenylpyrrolidine catalyst in the presence of α,β-unsaturated aldehyde, silyloxyfuran, 4-NBA and DCM to achieve the common intermediate γ-butenolide in 68% yield with excellent enantioselectivity (91% *ee*). Further transformations afforded the abovementioned four tricyclic polyketides via a three to eight steps sequence including a biomimetic [2+2] photocycloaddition, and one-pot oxidative cleavage/Baeyer-Villiger rearrangement or an unprecedented hydrogen-atom-transfer (HAT)-triggered oxygenation.

**Scheme 21:**
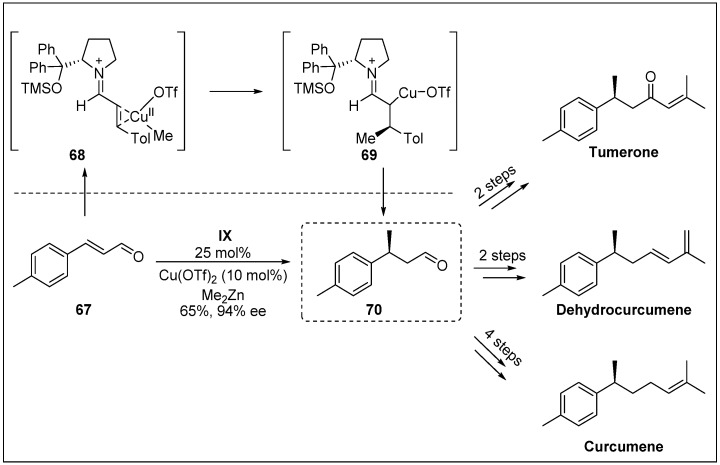
Total synthesis of (+)-dehydrocurcumene, (+)-tumerone, and (+)-curcumene.

In 2011, the Córdova group [60] accomplished the enantioselective total syntheses of three diverse bisabolane sesquiterpenes, namely, (+)-dehydrocurcumene, (+)-tumerone, and (+)-curcumene starting from the common precursor **70**, which was prepared via co-catalytic asymmetric conjugate addition (Scheme 21).

This asymmetric addition highlighted the use of catalyst IX in combination with Cu(OTf)_2_ in the presence of α,β-unsaturated aldehyde **67**, dimethylzinc and THF to achieve 1,4-selective product **70** in 65% yield with excellent enantioselectivity (94% *ee*) via Cu(II)-alkyl complex **68** and subsequent selective 1,4-addition. Further transformations afforded the above three sesquiterpenes by a two to four steps sequence from the common precursor **70**.

**Scheme 22:**
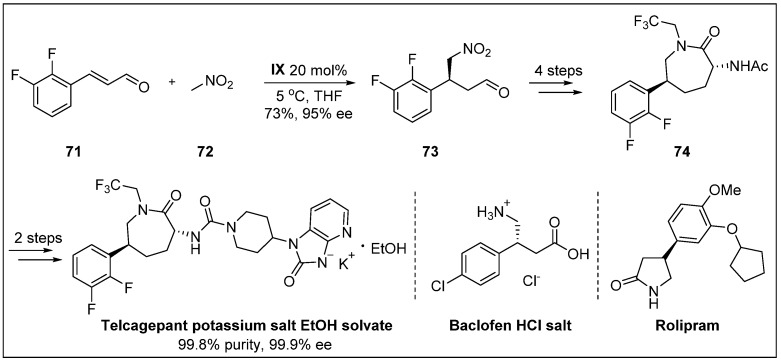
Total synthesis of MK-0974, baclofen, and rolipram.

In 2010, the Xu group [61] reported an efficient total synthesis of a selective peptide receptor antagonist telcagepant (MK-0974) (Scheme 22), which is a migraine drug [62] developed by Merck & Co Inc.

This key sequence highlighted the use of Jorgensen’s catalyst IX in the presence of α,β-unsaturated aldehyde **71**, nitromethane (**72**), pivalic acid, boric acid and THF to give adduct **73** in 73% yield with excellent enantioselectivity (95% *ee*). The advanced precursor **74** was then obtained by a four-step sequence including a formal Doebner-Knoevenagel coupling, reduction and cyclization. Further transformations afforded telcagepant potassium salt EtOH solvate with near perfect enantioselectivity (99.9% *ee*). It is interesting to note that this is the first reported application case from laboratory scale to industrial scale, which shows the great potential of asymmetric iminium catalysis.

In addition, the Wang group [63] performed a 3-step total synthesis of the antispastic drug baclofen with 97% *ee* using the same strategy. Interestingly, the Palomo group [64] reported a 2-step total synthesis of type IV phosphodiesterase inhibitor (+)-rolipram via a new chiral secondary amine VIII using water as the only solvent.

**Scheme 23:**
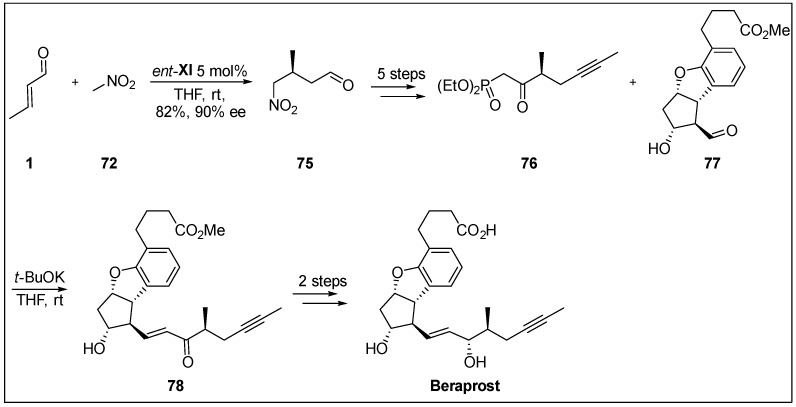
Total synthesis of (+)-beraprost.

Nitromethane is one of the most prominent nucleophiles used in Michael addition reactions. In 2017, the Hayashi group [65] successfully developed an excellent total synthesis of the most biologically active isomer (+)-beraprost (Scheme 23), which is a prostaglandin-like drug developed by Toray Industries Inc. [66]. This FDA-approved agent contains a unique benzofuran ring system and four contiguous stereogenic centers.

As depicted in Scheme 23, mechanistically, the introduction of the tertiary stereocenter could be easily furnished by reacting crotonaldehyde (**1**) with nitromethane (**72**) in the presence of Jorgensen’s catalyst *ent*-XI. This strategy gave adduct **75** in 82% yield with excellent enantioselectivity (90% *ee*).

Further transformations afforded phosphonate **76** by a five-step sequence including an Ohira-Bestmann reaction, selective methylation, Nef reaction, benzyl esterification, and Claisen-type reaction. Another three-step transformation including a Horner-Wadsworth-Emmons reaction, diastereoselective 1,2-reduction, and subsequent hydrolysis afforded (+)-beraprost.

**Scheme 24:**
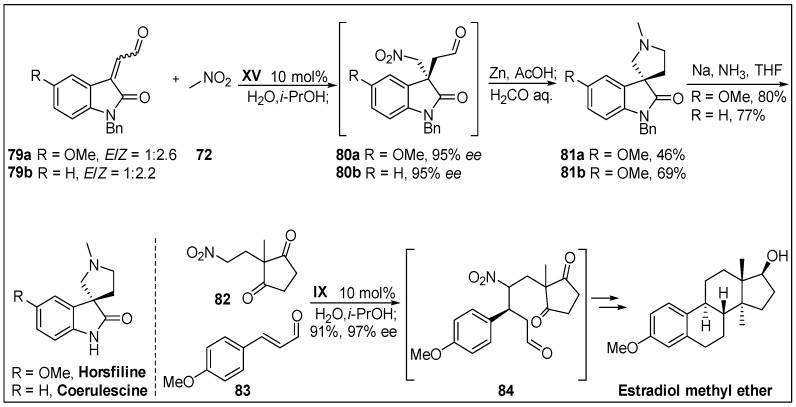
Total synthesis of (−)-horsfiline, (−)-coerulescine, and estradiol methyl ether.

In 2014, the Hayashi group [67] accomplished an enantioselective total synthesis of spirooxyindole alkaloids (−)-horsfiline and (−)-coerulescine (Scheme 24). (−)-Horsfiline was first isolated from *Horsfieldia superba* in 1991 by the Bodo group [68], while (−)-coerulescine was isolated from *Pharalis coerulescens* in 1998 by the Colegate group [69].

The key step in this elegant total synthesis was a highly enantioselective one-pot Michael addition/reductive amination/reductive amination sequence for the construction of the all-carbon quaternary stereogenic centers with excellent enantioselectivity. After the abovementioned one-pot four-reaction process, one further transformation afforded (−)-horsfiline and (−)-coerulescine. Another impressive total synthesis using this type of method was accomplished by the Hayashi group [70] in 2017. In their synthesis of estradiol methyl ether, the key step was a chiral secondary amine IX catalyzed Michael addition of nitroalkane **82** and cinnamaldehyde **83** (Scheme 24).

### 2.3. Hydrogenation

**Scheme 25:**
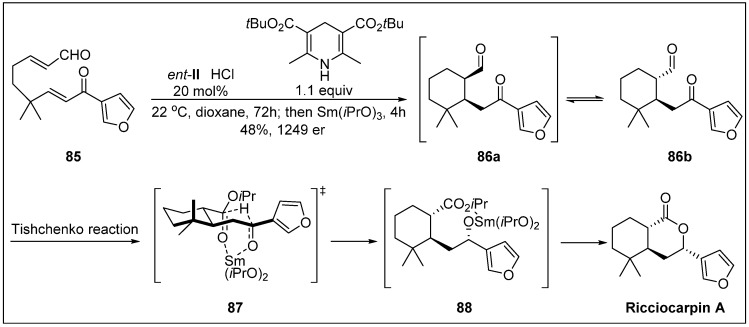
Total synthesis of (+)-ricciocarpin A.

In 2009, the List group [71] develop a novel method for the perfectly redox-economic total synthesis of the furanosesquiterpene lactone (+)-ricciocarpin A (Scheme 25), which had been isolated from *Ricciocarpos natans* [72] and exhibited potent molluscicidal activity [73]. The above group employed a cascade reductive Michael/cycloisomerization/Tishchenko reaction for the rapid construction of its chiral centers.

The synthetic precursor of the key organocatalytic reaction was obtained via an aldol condensation and cross-metathesis. Thus, when α,β-unsaturated aldehyde **85** was treated with catalyst *ent*-II as well as Hantzsch ester in the presence of dioxane at 22 °C, the reductive Michael reaction initially proceeded in a highly enantioselective (97% *ee*) manner to provide ketoaldehyde **86a**. Remarkably, isomerization and Tishchenko reaction occurred rapidly upon treating the above reaction mixture with Sm(*i*PrO)_3_. This protecting-group-free 3-step total synthesis of (+)-ricciocarpin A shows the great potential of asymmetric iminium catalysis.

**Scheme 26:**
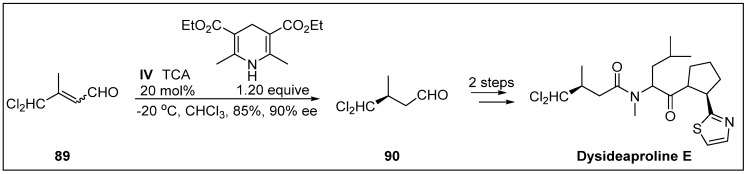
Total synthesis of (+)-dysideaproline E.

In 2011, the Willis group [74] reported the first total synthesis of polychlorinated compound (+)-dysideaproline E (Scheme 26), which had been isolated from marine sponge *Dysidea* sp. in 2001 by Harrigan group [75]. The Willis group employed an organocatalytic reductive Michael reaction to construct its chiral center.

Synthetic precursor **89** of the key organocatalytic reaction was obtained in two steps via a Horner-Wadsworth-Emmons reaction and DIBAL-H reduction. Thus, when α,β-unsaturated aldehyde **89** was treated with catalyst IV as well as Hantzsch ester in the presence of CHCl_3_ at −20 °C, the reductive Michael proceeded in a highly enantioselective (90% *ee*) to achieve dichloroaldehyde **90**. Two further high yielding steps then gave (+)-dysideaproline E in an efficient and highly rapid procedure.

### 2.4. Cycloadditions

In comparison to the above conjugated addition, organocatalytic cycloaddition strategies also provide an effective methodology to produce key chiral intermediates for the construction of important natural products and drugs [76]. The related cycloaddition strategies mentioned below including cyclopropanations, epoxidations, Diels-Alder reactions, [3+2]-cycloadditions, and [3+3]-cycloadditions.

#### 2.4.1. Cyclopropanations

Chiral tricyclic compounds are very important precursors in total synthesis of bioactive natural products, as well as useful building blocks of medicinal synthesis chemistry. Organocatalytic cyclopropanation reactions provide an important route for the construction of the key three-membered ring precursors. To illustrate the substantial potential of this methodology, three very interesting examples for the application of chiral secondary amine-catalyzed reactions will be discussed.

**Scheme 27:**
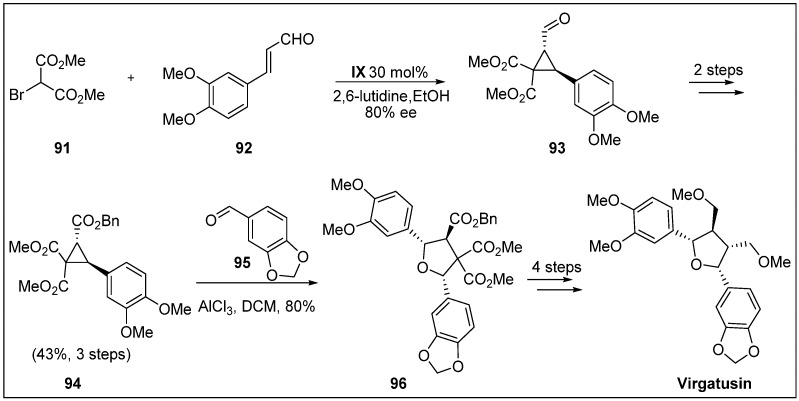
Total synthesis of (+)-virgatusin.

In 2009, the Johnson group [77] developed a novel method for the enantioselective total synthesis of the tetrahydrofuran lignan (+)-virgatusin (Scheme 27), which had been first isolated by the Chen group [78] in 1996 from the Chinese herbal medicine *Phyllantus virgatus* and exhibited antifungal activity [79]. The above group employed an intermolecular enantioselective organocatalytic cyclopropanation reaction for the construction of its incipient chiral centers.

This key sequence highlighted the use of Jorgensen’s catalyst IX in the presence of α,β-unsaturated aldehyde **92**, bromomalonate **91**, 2,6-lutidine and EtOH togeneratecyclopropane **93** in 80% *ee* via a tandem Michael addition/enamine-activated α-alkylation reaction. Further transformations afforded (+)-virgatusin by a seven-step sequence including AlCl_3_-catalyzed [3+2]-cycloaddition. Notably, after single recrystallization of **96**, optical purity was improved to 98% *ee*.

**Scheme 28:**
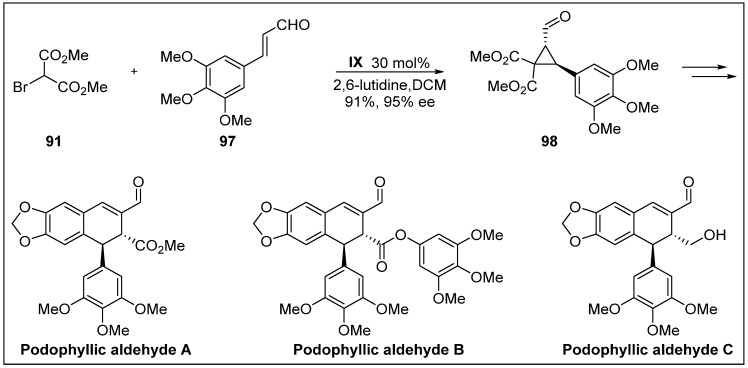
Total synthesis of (+)-podophyllic aldehydes A–C.

In 2014, the Nishii group [80] published the first enantioselective total synthesis of dihydronaphthalene lignans (+)-podophyllic aldehydes A, B and C (Scheme 28), which exhibited notable antineoplastic activity [81]. The key reaction in this total synthesis was a highly enantioselective tandem Michael/alkylation reaction also using organocatalytic cascade strategy.

This key sequence highlighted the use of Jorgensen’s catalyst IX in the presence of α,β-unsaturated aldehyde **97**, bromide **91**, 2,6-lutidine and DCM to achieve cyclopropane **98** in 91% yield with 95% *ee* via tandem Michael addition/enamine-activated α-alkylation reaction. Further transformations afforded (+)-podophyllic aldehydes A, B and C by an eight to fifteen steps sequence including BF_3_·Et_2_O-mediated chiral transfer ring expansion.

**Scheme 29:**
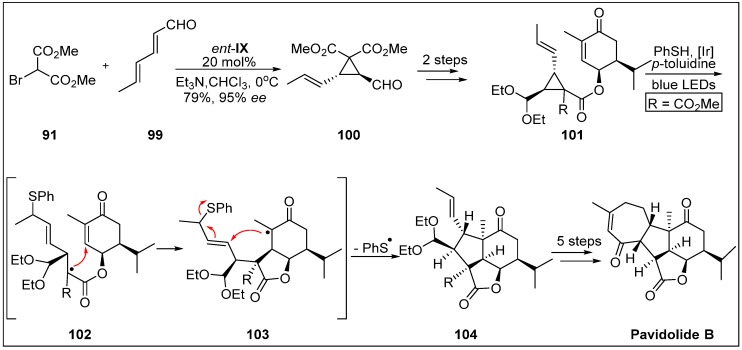
Total synthesis of (−)-pavidolide B.

Pavidolide B is a tetracyclic diterpenoid isolated from the soft coral *Sinularia pavida* in 2012 by the Lin group [82] that displays highly selective inhibition against the human cancer cell line HL-60. This compound contains an unprecedented 6,5,7-tricarbocyclic core structure which includes a synthetically challenging fully functionalized cyclopentane and seven contiguous stereogenic centers. In 2017, an asymmetric total synthesis of (-)-pavidolide B was elegantly disclosed by Yang group [83] via an enantioselective organocatalytic cyclopropanation reaction to build the incipient chiral centers (Scheme 29).

This key sequence highlighted the use of Jorgensen’s catalyst *ent*-IX in the presence of α,β,γ,δ-unsaturated aldehyde **99**, bromide **91**, Et_3_N and THF to achieve cyclopropane **100** in excellent enantioselectivity (95% *ee*) via tandem intermolecular Michael addition/intramolecular α-alkylation reaction. Further transformations afforded (−)-pavidolide B by a nine-step sequence including intramolecular radical annulation, Ni-catalyzed cross-coupling reaction, ring-closing metathesis reaction, and RhCl_3_-catalyzed double bond isomerization. It is worth mentioning that the highly efficient total synthesis afforded (−)-pavidolide B on a 300 mg scale and provided a good foundation for its further biological investigation.

#### 2.4.2. Epoxidations

**Scheme 30:**
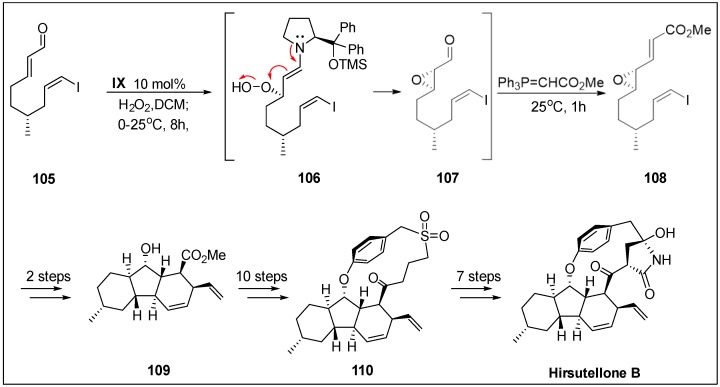
Total synthesis of (+)-hirsutellone B.

In 2005 the Jørgensen group introduced a novel strategy for the asymmetric organocatalytic epoxidation of α,β-unsaturated aldehydes using H_2_O_2_ as the oxidant. Using this strategy, the Nicolaou group [84] described an excellent total synthesis of (+)-hirsutellone B (Scheme 30), which had been first isolated from fungus *Hirsutella nivea* BCC 2594 in 2005 by Isaka group [85] and exhibited antibiotic activity. The Nicolaou group employed an enantioselective one-pot epoxidation/Wittig reaction to construct its initial chiral center.

**Scheme 31:**
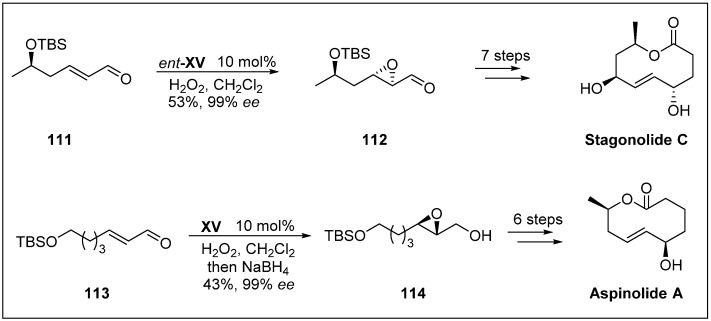
Total synthesis of (+)-stagonolide C and (-)-aspinolide A.

Synthetic precursor **105** of the key organocatalytic reaction was prepared by a three-step transformation from known (+)-citronellal. Thus, when α,β-unsaturated aldehyde **105** was treated with 10 mol% of catalyst IX in the presence of H_2_O_2_, the asymmetric epoxidation proceeded in a highly enantioselective manner. Mechanistically, nucleophilic attack of the enamine intermediate **106** at the electrophilic peroxygen atom to give an α,β-epoxidized iminium intermediate **107**. Then the resulting aldehyde **107** was converted directly into the epoxy ester iodide **108** via Wittig reaction. Further transformations afforded (+)-hirsutellone B by a nineteen-step sequence including cascade intramolecular epoxide opening/[4+2] cycloaddition reaction, Barton etherification and Ramberg-Bäcklund reaction.

(+)-Stagonolide C was isolated in 2007 from *Stagonospora cirsii* [86], while (−)-aspinolide A was isolated in 1997 from *Aspergillus ochraceus* [87]. In 2012, The Sudalai group [88] reported the enantioselective total synthesis of the above two 10-membered lactones (+)-stagonolide C and (−)-aspinolide A via a similar organocatalytic epoxidation process (Scheme 31).

**Scheme 32:**
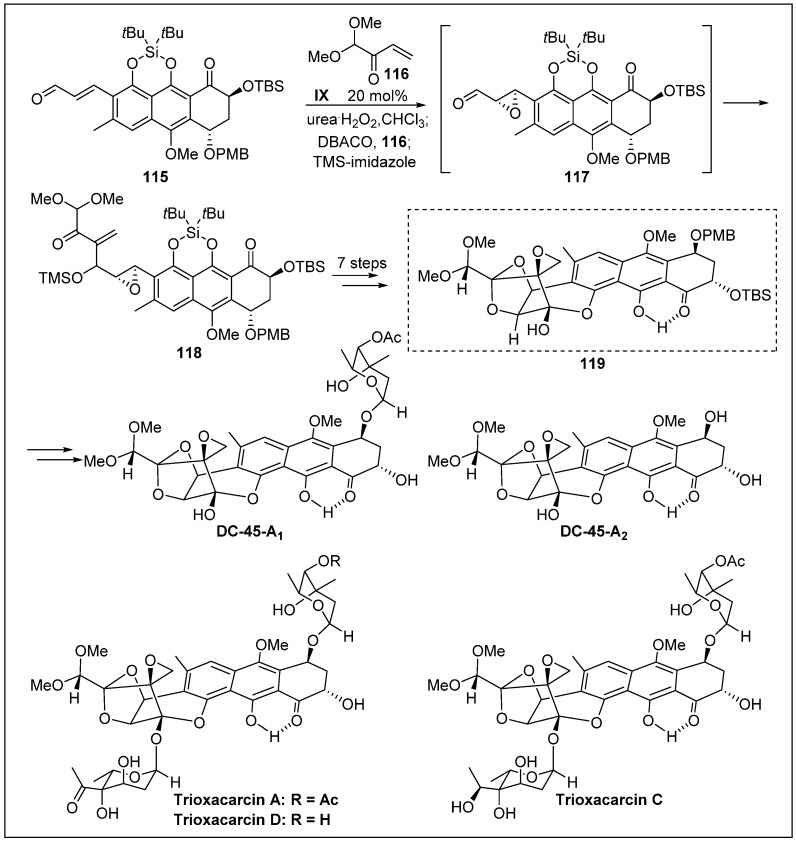
Total synthesis of trioxacarcins DC-45-A_1_, A_2_, A, C and D.

Between 2015 and 2017, the Nicolaou group [89,90,91] developed the elegant divergent total synthesis of five highly potent cytotoxic agents trioxacarcins DC-45-A1, DC-45-A2, A, C, and D from a common trioxacarcin precursor **119**, which was efficiently constructed using asymmetric organocatalytic epoxidation as one of the most versatile strategies (Scheme 32).

Synthetic precursor **115** of the key epoxidation reaction was prepared by a twelve-step transformation from cyclohexadiene. Then the key sequence highlighted the use of Jorgensen’s catalyst IX in the presence of α,β,-unsaturated aldehyde**115**, urea·H_2_O_2_, and CHCl_3_/H_2_O (20:1) to achieve epoxyaldehyde **117**, which gave epoxyketone **118** via a one-pot Baylis-Hillman reaction of **117** followed by TMS-protection reaction. With epoxyketone **118** in hand, further transformations provided common anthraquinone precursor **119** on 200 mg scale by a seven-step sequence including BF_3_·OEt_2_-catalyzed epoxyketone rearrangement, Upjohn dihydroxylation, TPAP-catalyzed oxidation, and selectively deprotected. Compound **119** then served as the key common precursor for the successful total synthesis of above five anthraquinone trioxacarcins by a two to seven steps sequence.

**Scheme 33:**
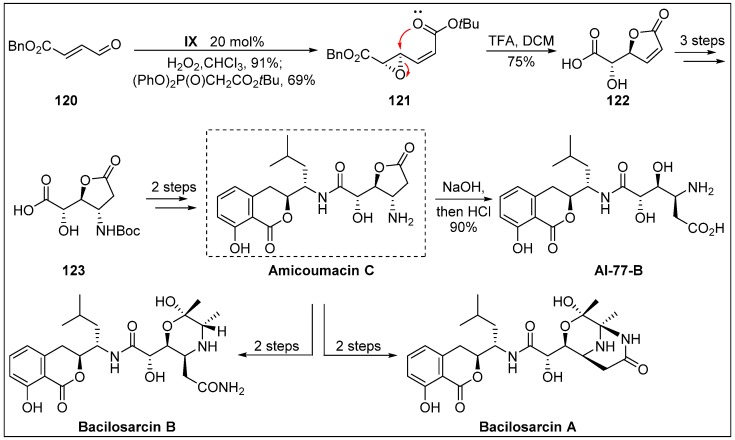
Total synthesis of (-)-bacilosarcins A, B and (-)-AI-77-B.

In 2009, the Kuwahara group [92] reported the first enantioselective total synthesis of isocoumarins (−)-bacilosarcins A and B (Scheme 33), which had been isolated in 2008 by the Igarashi group [93] from a marine *Bacillus subtilis* bacterium and exhibited growth inhibition against millet. The group employed a highly enantioselective epoxidation reaction for the construction of its initial chiral building block by taking advantage of iminium activation strategy.

This key sequence highlighted the use of Jorgensen’s catalyst IX in the presence of α,β-unsaturated aldehyde **120**, H_2_O_2_ and CHCl_3_ to achieve enantiomerically pure epoxide. Further transformations provided common precursor amicoumacin C by a seven-step sequence including intramolecular epoxide ring-opening reaction, and hydrogenolysis. The common amide intermediate amicoumacin C was then successfully converted further either into (−)-bacilosarcins A or B by a two-step sequence. In addition, they also reported another new efficient total synthesis of (−)-AI-77-B.

**Scheme 34:**
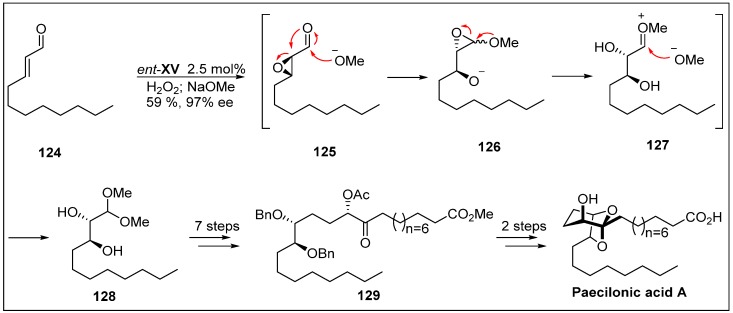
Total synthesis of (+)-paecilonic acid A.

In 2017, the Fürstner group [94] reported the first enantioselective total synthesis of bicyclic fatty acid (+)-paecilonic acid A (Scheme 34), which had been isolated from *Paecilomyces varioti* in 2016 by the Jung group [95] and possessed a unique 6,8-dioxabicyclo [3.2.1]octane core structure. The group employed an organocatalytic *trans*-dihydroxylation reaction [96] to construct two contiguous chiral centers.

The key sequence highlighted the use of Jorgensen’s catalyst *ent*-XV in the presence of α,β-unsaturated aldehyde **124**, H_2_O_2_, DCM, NaOMe and MeOH to achieve 1,2-diol **128** on scale with excellent enantioselectivity (97% *ee*) via one-pot epoxidation/ring opening reaction. Further transformations afforded acyloin **129** on 2 g scale by a seven-step sequence including benzylation, Wittig reaction, asymmetric alkynylation, and ruthenium-catalyzed *trans*-hydrostannation. Further transformations afforded (+)-paecilonic acid A by a simple two-step sequence including one-pot hydrogenolysis and acetalation as well as subsequent saponification.

#### 2.4.3. Diels-Alder Reactions

Undoubtedly, Diels-Alder reactions have played a dominant role in the area of total synthesis of drugs and biologically important products for many years. The interest in this methodology has increased in the last few years, especially for chiral secondary amines catalyzed enantioselective Diels-Alder reactions [1].

**Scheme 35:**
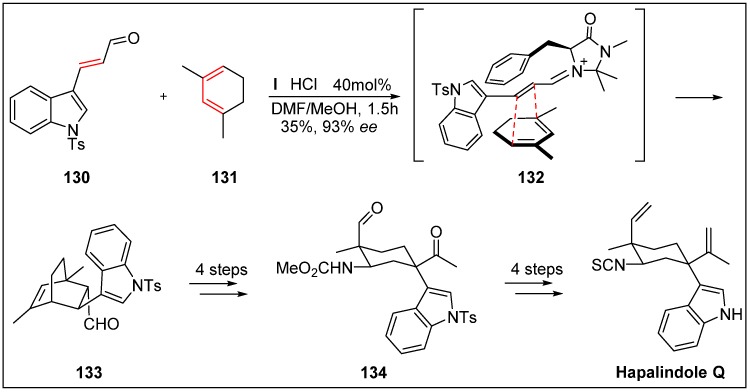
Total synthesis of (+)-hapalindole Q.

In 2003, the Kerr group [97] reported an excellent total synthesis of the tricyclic indole alkaloid (+)-hapalindole Q (Scheme 35), which had been first isolated from *Hapalosiphon fontinalis* [98] and exhibited antialgal as well as inhibit RNA polymerase activities [99]. The group employed an unprecedented intermolecular enantioselective [4+2] cycloaddition for the construction of its four consecutive stereocenters including an all-carbon quaternary stereogenic center.

This key sequence highlighted the use of MacMillan’s catalyst I in the presence of α,β-unsaturated aldehyde **130**, diene **131** and a mixed DMF/MeOH (1:1) solvent system containing 5% water to achieve cycloadduct **133** via the iminium complex **132** in 35% yield with excellent enantioselectivity (93% *ee*). Then keto-aldehyde **134** was obtained through a sequence including oxidation of the aldehyde, Curtius rearrangement, dihydroxylation, and diol cleavage. The final transformation from **134** to (+)-hapalindole Q was achieved by a sequential Wittig olefination and double deprotection. Notably, this is not only the first impressive example of total synthesis using MacMillan’s intermolecular Diels-Alder strategy, but also showed the great potential of asymmetric iminium catalysis.

**Scheme 36:**
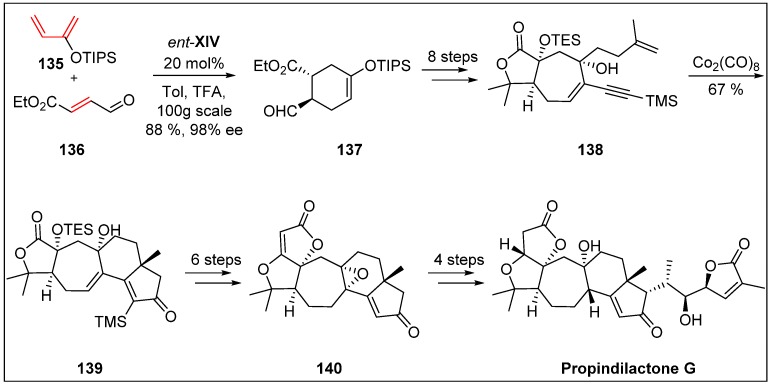
Total synthesis of (+)-propindilactone G.

In 2015, the Yang group [100] accomplished the first enantioselective total synthesis of nortriterpenoid (+)-propindilactone G (Scheme 36), which had been isolated from *Schisandra propinqua* var. *propinqua* and exhibited anti-HIV activity [101]. The group employed an intermolecular highly enantioselective Diels-Alder reaction for the construction of its chiral building block by taking advantage of iminium activation strategy.

This key sequence highlighted the use of Hayashi-Jorgensen’s catalyst XIV in the presence of α,β-unsaturated aldehyde **136**, diene **135**, TFA and toluene to achieve cycloadduct **137** with superb enantioselectivity (98% *ee*). Final assembly of the different building units gave rise to (+)-propindilactone G by a nineteen-step sequence including Co-mediated Pauson-Khand reaction, Pd-catalyzed reductive hydrogenolysis reaction and OsO_4_-mediated regio- and stereoselective dihydroxylation. It is interesting to note that the key asymmetric Diels-Alder reaction not only provided a good yield (88% yield in 100 g scale) in the total synthesis but indicated its efficiency in the construction of stereocenters (98% *ee*).

**Scheme 37:**
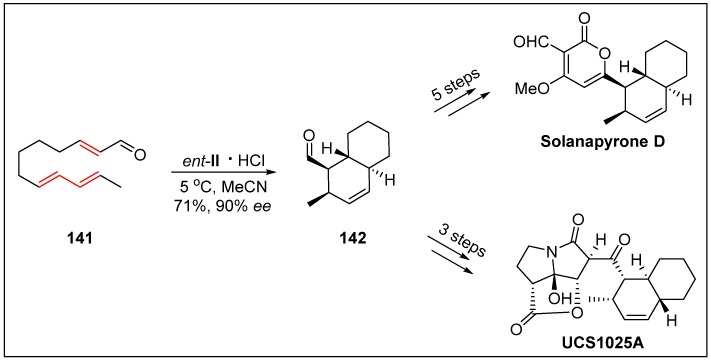
Total synthesis of (-)-solanapyrone D and (+)-UCS1025A.

In 2005, the MacMillan group [102] described the first organocatalytic intramolecular Diels-Alder reaction and demonstrated a concise total synthesis of phytotoxic polyketide (−)-solanapyrone D (Scheme 37), which had been isolated from *Altenaria solani* [103]. The key reaction in this elegant synthesis was an intramolecular highly enantioselective Diels-Alder reaction using their group newly established iminium activation strategy.

It is noteworthy that all four consecutive stereocenters of the above product was achieved in 71% yield through the use of their own catalyst *ent*-II in the presence of α,β-unsaturated aldehyde **141** and the solvent of MeCN. Cycloadducts **142** was then converted successfully into (-)-solanapyrone D in a series of highly efficient transformations. The above asymmetric Diels-Alder reaction strategy has proven to be an extremely powerful tool for the total synthesis of complex molecules (6 steps total synthesis of (−)-solanapyrone D by the MacMillan group, vs. 19 steps total synthesis of (±)-solanapyrone D by the Hagiwara group [104]). In addition, the Danishefsky group [105] reported the concise total synthesis of (+)-UCS1025A using the common building block (Scheme 37).

**Scheme 38:**
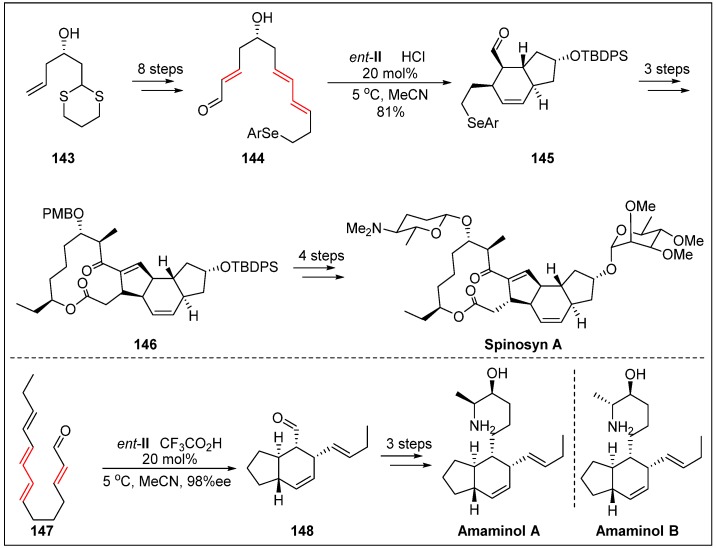
Total synthesis of (−)-spinosyn A, amaminols A, and B.

Spinosyn A is a tetracyclic-macrolide isolated from soil microorganisms *Saccharopolyspora spinosa* in 1991 by the Kirst group [106] that displays strong insecticidal activity. This FDA-approved agent contains a unique 5,6,5,12-fused tetracyclic core structure, which includes a synthetically challenging *trans*-5,6-fused ring and 5,12-fused macrolactone. The most recent convergent total synthesis of (-)-spinosyn A was elegantly disclosed in 2016 by the Dai group [107] who used an organocatalytic intramolecular Diels-Alder reaction developed by the MacMillan group to build the *trans*-5,6-fused ring in excellent diastereoselectivity (Scheme 38).

Synthetic precursor **144** of the key organocatalytic reaction was prepared by an eight-step transformation from known thioketal **143**. The key *trans*-5,6-fused ring intermediate **145** was obtained in 81% yield, and highly enantioselective was observed by treatment of α,β-unsaturated aldehyde **144** with 20 mol% of catalyst *ent*-II in the presence of MeCN at 5 °C. Further transformations afforded (−)-spinosyn A by a seven-step sequence including Au-catalyzed rearrangement, Pd-catalyzed carbonylative Heck macrolactonization and Au-catalyzed Yu glycosylation. Moreover, the Koskinen group [108] and the Christmann group [109] reported the total synthesis of amanimol A and amanimol B respectively, utilizing the same strategy of intramolecular Diels-Alder reaction (Scheme 38).

**Scheme 39:**
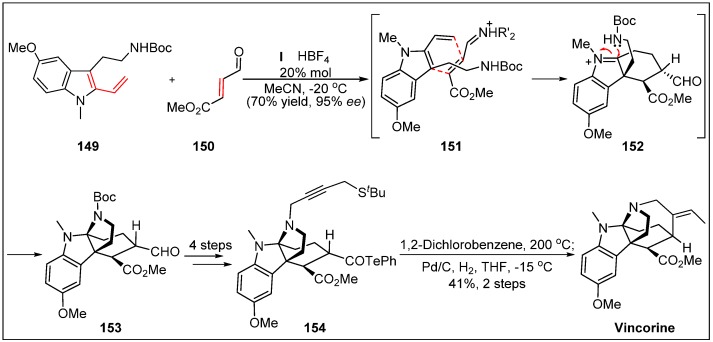
Total synthesis of (-)-vincorine.

In 2013, the MacMillan group [110] described the elegant and impressive nine-step total synthesis of an akuammiline alkaloid (−)-vincorine (Scheme 39), utilizing a similar organocatalytic cascade strategy. This key sequence highlighted the use of MacMillan’s catalyst I in the presence of α,β-unsaturated aldehyde **150** and diene **149** to achieve tetracyclic intermediate **153** in excellent enantioselectivity (95% *ee*) via intramolecular iminium cyclization of **152**, which was assembled by employing a stereoselective Diels-Alder reaction. Further transformations afforded (−)-vincorine by a six-step sequence including Pinnick oxidation, reductive amination, and 7-*exo*-dig radical cyclization. It is worth mentioning that this organocatalytic cascade strategy has proven to be an extremely effective tool.

#### 2.4.4. [3+2]-Cycloadditions

**Scheme 40:**
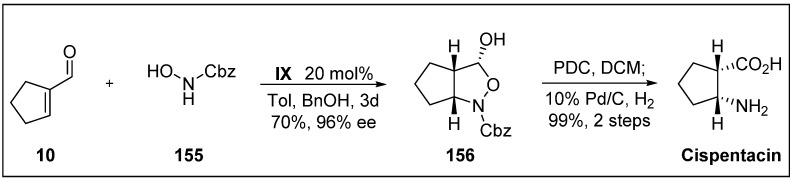
Total synthesis of cispentacin.

In 2013, the Moyano group [111] developed an excellent 3-step total synthesis of cispentacin (Scheme 40), which had been first isolated in 1989 from *Bacillus cereus* and exhibited antifungal activity [112]. The group employed an intermolecular highly enantioselective formal [3+2] cycloaddition for the construction of its two consecutive stereocenters.

This key sequence highlighted the use of Hayashi’s catalyst IX in the presence of α-branched α,β-unsaturated aldehyde **10**, *N*-Cbz-hydroxylamine **155**, BnOH and toluene to achieve isoxazolidine **156** in 70% yield with superb enantioselectivity (98% *ee*) via tandem *aza*-Michael addition/cyclization. Further transformations afforded cispentacin by a simple two-step sequence including PDC oxidation and subsequent catalytic hydrogenation.

**Scheme 41:**
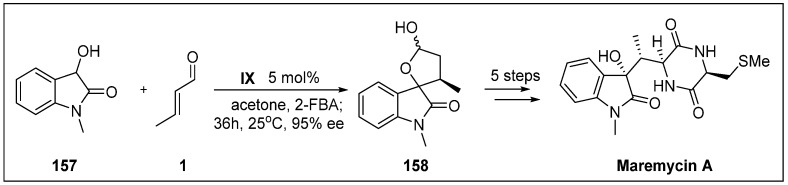
Total synthesis of (-)-maremycin A.

In 2012, the Melchiorre group [113] accomplished the novel enantioselective total synthesis of diketopiperazine alkaloid (−)-maremycin A (Scheme 41), which was isolated from *Streptomyces* species B 9173 [114]. The key reaction in this elegant total synthesis was a highly enantioselective domino Michael addition/lactol cyclization reaction using dioxindole as a nucleophile.

This key sequence highlighted the use of Jørgensen’s catalyst IX in the presence of α,β-unsaturated aldehyde **1**, 3-substituted 3-hydroxyoxindole **157**, acetone and *ortho*-fluorobenzoic acid to achieve lactol **158** on a gram scale with excellent enantioselectivity (95% *ee*). Further transformations afforded (−)-maremycin A by a five-step sequence including PCC oxidation and Staudinger reaction.

#### 2.4.5. [3+3]-Cycloadditions

**Scheme 42:**
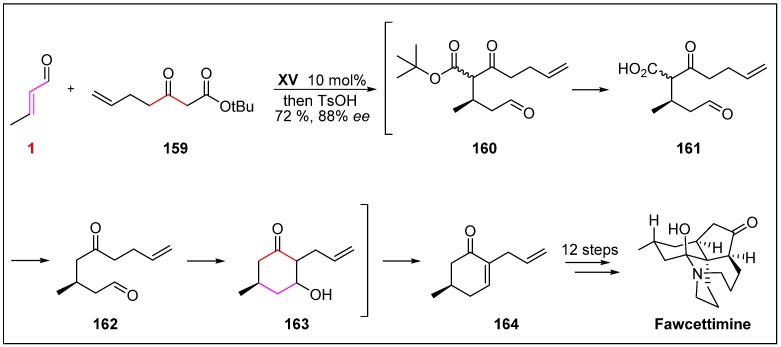
Total synthesis of (+)-fawcettimine.

In 2007, the Toste group [115] accomplished an enantioselective total synthesis of tetracyclic alkaloid (+)-fawcettimine (Scheme 42), which was first isolated in 1959 from *Lycopodium* by the Burnell group [116]. The key reaction in this elegant total synthesis was a highly enantioselective one-pot Michael addition/cyclization process. This is the first reported case of total synthesis via organocatalytic conjugated addition of β-ketoesters to α,β-unsaturated aldehydes [117].

This asymmetric Robinson annulation sequence highlighted the use of Jorgensen’s catalyst XV in the presence of crotonaldehyde (**1**), ketoester **159**, *p*-TsOH and toluene to produce dienone **164** on 10 g scale with excellent enantioselectivity (88% *ee*) [118]. Formation of **164** can be explained by an initial Michael addition, followed by a decarboxylation of the ester **160**, and a final intramolecular aldol condensation of the aldehyde **162**. Further transformations afforded (+)-fawcettimine by a twelve-step sequence including conjugate propargylation, Au(I)-catalyzed cyclization, Pd-catalyzed cross-coupling, intramolecular S_N_2 reaction and Dess-Martin oxidation.

**Scheme 43:**
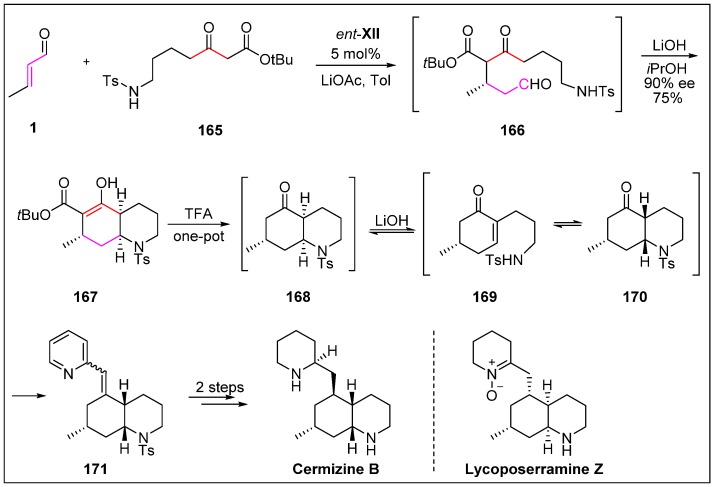
Total synthesis of (-)-cermizine B and (+)-lycoposerramine Z.

In 2014, the Bradshaw group [119] accomplished a gram-scale total synthesis of *cis* phlegmarane alkaloid (−)-cermizine B (Scheme 43), which first isolated from *Lycopodium cernuum* by Kobayashi group [47] in 2004.

This key sequence highlighted the use of Jorgensen’s catalyst ent-XII in the presence of aldehyde **1**, β-keto ester **165**, LiOAc, LiOH and toluene to achieve azabicyclic intermediate **167** with 90% *ee* via a one-pot tandem Michael addition/Robinson annulation/intramolecular *aza*-Michael addition. Furthermore, an elegant 10-step total synthesis of (+)-lycoposerramine Z via this methodology was reported in 2013 by the same group [120].

**Scheme 44:**
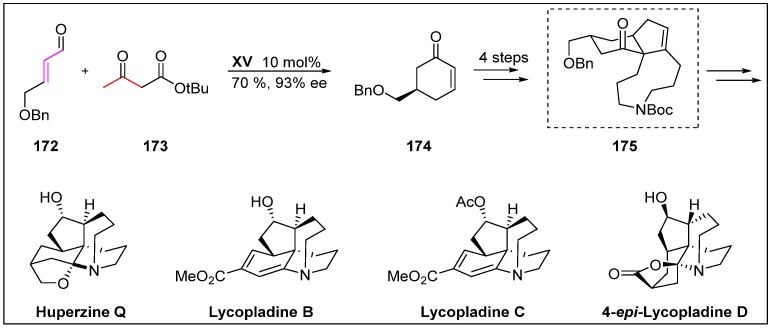
Total synthesis of (−)-huperzine Q, (+)-lycopladine B, and (+)-lycopladine C.

A similar method was recently reported by the Lei group [121] who used an iminium-catalyzed Robinson annulation reaction to build up the required skeleton enone **174** of *Lycopodium* alkaloids, namely, (−)-huperzine Q, (+)-lycopladine B, (+)-lycopladine C, and (−)-4-*epi*-lycopladine D (Scheme 44) starting from the common precursor **175**.

Synthetic precursor **175** was prepared by simply four-step transformation from enone **174**, which was prepared via an unprecedented organocatalytic asymmetric five-step one-pot procedure. This asymmetric Robinson annulation sequence highlighted the use of Jorgensen’s catalyst XV in the presence of α,β-unsaturated aldehyde **172**, *tert-*butyl-3-oxobutyric ester **173**, *p*-TsOH and toluene to afford cyclohexenone **174** in 70% yield with excellent enantioselectivity (93% *ee*) via tandem Michael addition/hydrolyzation/decarboxylation/aldol condensation reaction. Further transformations afforded the aforementioned four alkaloids by a six to seven steps sequence from the common precursor **175**.

**Scheme 45:**
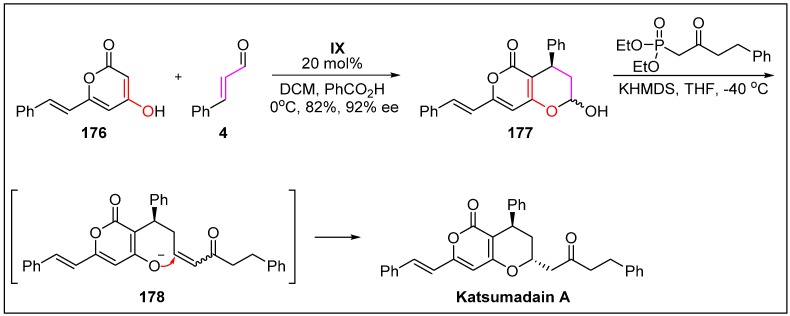
Total synthesis of (-)-katsumadain A.

In 2013, the Tang group [122] accomplished the first total synthesis of influenza virus neuraminidase inhibitor (−)-katsumadain A (Scheme 45), which had been isolated from the Chinese herbal medicine *Alpinia katsumadai Hayata* [123] and exhibited antiemetic activity. The above group employed an organocatalytic enantioselective 1,4-conjugate addition for the construction of its benzylic chiral centers.

This sequence involved the use of Jorgensen’s catalyst IX in the presence of α,β-unsaturated aldehyde **4**, pyranone **176**, benzoic acid and DCM to achieve lactol 1**77** in 92% *ee* via tandem intermolecular Michael addition/intramolecular cyclization. Further transformations afforded (−)-katsumadain A by a tandem Horner-Wadsworth-Emmons reaction/*oxa*-Michael addition.

**Scheme 46:**
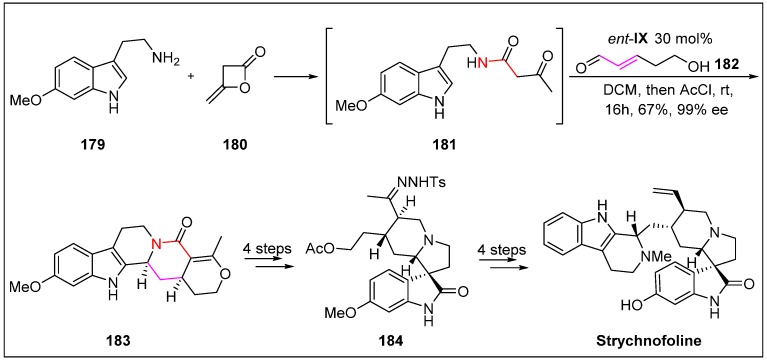
Total synthesis of (-)-strychnofoline.

Very recently, the Xu group [124] accomplished the first enantioselective nine-step total synthesis of (-)-strychnofoline (Scheme 46), an anticancer [125] alkaloid containing a unique spirooxindole architecture, which was isolated in 1978 from the leaves of *Strychnos usambarensis* [126]. The key reaction in this elegant total synthesis was a highly enantioselective domino reaction using iminium activation strategy.

The key organocatalytic asymmetric transformation included a one-pot, three-component reaction between diketene **180**, 6-methoxytryptamine **179**, and acrolein derivative **182** to afford the quinolizidine intermediate **183** with superb enantioselectivity (99% *ee*). This remarkable transformation can be rationalized by a cascade sequence involving an acylation giving intermediate **181** first, followed by a Michael addition and a final Pictet-Spengler reaction to yield the intermediate **183**. Then hydrazone **184** was obtained through a sequence including oxidative rearrangement, selective amide reduction, hydrolysis, acylation and the transformation of ketone. Final transformation from **184** to (−)-strychnofoline was achieved by a sequential Shapiro tosylhydrazone decomposition, Dess-Martin oxidation, Pictet-Spengler reaction and demethylation.

**Scheme 47:**
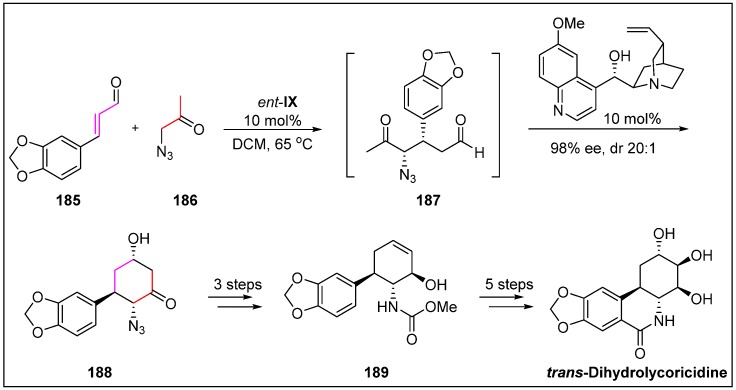
Total synthesis of (+)-*trans*-dihydrolycoricidine.

In 2014, the McNulty group [127] accomplished an enantioselective nine-step total synthesis of (+)-*trans*-dihydrolycoricidine (Scheme 47), an anticancer *Amaryllidaceae* alkaloid containing five contiguous stereogenic centers, which was isolated in 1993 from *Hymenocallis caribaea* by the Pettit group [128]. The key reaction in this elegant total synthesis was a highly enantioselective sequential [3+3]-type Michael/aldol reaction using cocatalyst strategy of secondary-amine *ent*-IX/quinidine.

This key sequence highlighted the use of cocatalyst *ent*-IX and quinidine in the presence of α,β-unsaturated aldehyde **185**, α-azido acetone **186** and DCM to achieve aminocyclitol ring **188** in 65% yield with 98% *ee* via tandem *syn* Michael addition/intramolecular aldol reaction. Further transformations afforded (+)-*trans*-dihydrolycoricidine by an eight-step sequence including reduction, epoxidation, and Bischler-Napieralski reaction.

**Scheme 48:**
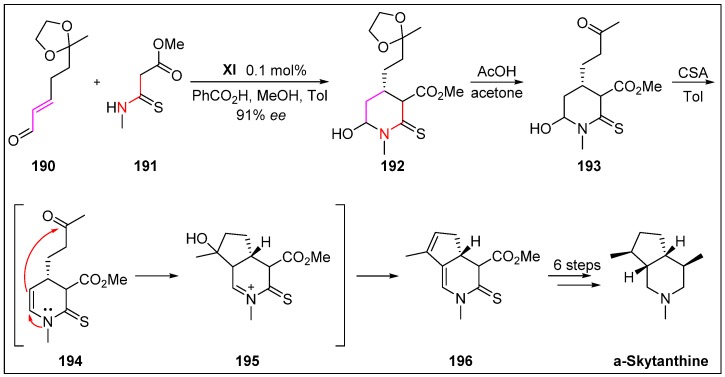
Total synthesis of (+)-*α*-skytanthine.

In 2015, the Ishikawa group [129] disclosed the enantioselective total synthesis of monoterpene piperidine alkaloid (+)-α-skytanthine (Scheme 48), which had been isolated from *Skytanthus actus* [130] and exhibited hypotensive activity [131]. The group employed a formal *aza*-[3+3] cycloaddition for the construction of piperidine ring **192** by taking advantage of iminium activation strategy.

This key sequence involved the use of Hayashi’s catalyst XI in the presence of α,β-unsaturated aldehyde **190**, *N*-methyl thiomalonamate **191**, benzoic acid, MeOH and toluene to achieve cycloadduct **192** in excellent enantioselectivity (91% *ee*) on gram-scale quantities. The successful synthesis of (+)-α-skytanthine could then be accomplished in eight more chemical steps including intramolecular aldol condensation, epimerization, and hydrogenation. It is interesting to note that the key cycloaddition reaction not only introduced all the carbons in one step but also indicated the incredible potential of Hayashi’s catalyst (at only 0.1 mol% loading).

**Scheme 49:**
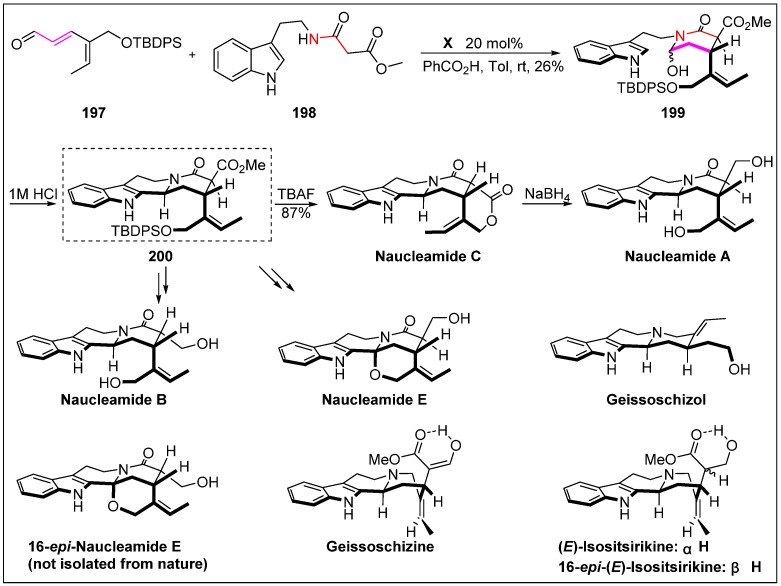
Total syntheses of eight monoterpenoid indole alkaloids.

In 2017, the Jia group [132] accomplished the enantioselective total syntheses of eight diverse monoterpenoid indole alkaloids (Scheme 49), namely, naucleamides A-C and E, geissoschizine, geissoschizol, 16-*epi*-(*E*)-isositsirikineand, and (*E*)-isositsirikine. The key asymmetric transformation featured a cascade Michael addition/Pictet-Spengler reaction using iminium activation strategy.

Synthetic precursor **197** of the key organocatalytic reaction was prepared by a simple four-step transformation. Then the key sequence used Hayashi-Jorgensen’s catalyst X in the presence of α,β-unsaturated aldehyde **197**, amidomalonate **198**, PhCO_2_H and toluene to achieve *E*-isomer **199**, which was cyclized with 1 M HCl to achieve common intermediate **200**. Final transformation from intermediate **200** to the above eight alkaloids were achieved by one to six step sequences.

### 2.5. Organocascade Reactions

Asymmetric cascade reactions [133] have attracted increasingly prominent interest over the last eighteen years as this novel synthetic strategy can facilitate the complex architectures of natural products in unprecedented synthetic efficiency with excellent enantioselectivity.

**Scheme 50:**
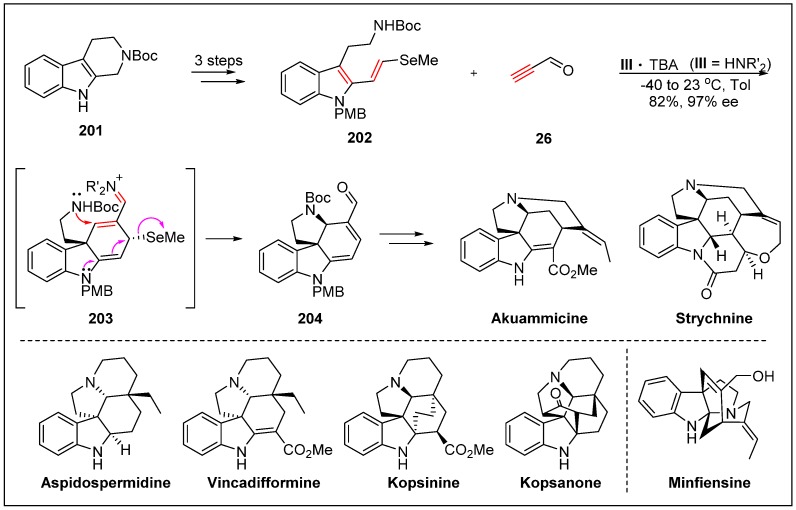
Total synthesis of (−)-strychnine, (+)-minfiensin and five other alkaloids.

In 2011, the MacMillan group [134] reported a novel methodology to stereoselectively synthesize both **203** and *ent*-**203** through a tandem intermolecular Diels-Alder/elimination/intramolecular Michael addition (Scheme 50). They efficiently applied their highly-potent strategy to the asymmetric total synthesis of six alkaloids, (−)-strychnine, (−)-akuammicine, (+)-aspidospermidine, (+)-vincadifformine, (−)-kopsinine, and (−)-kopsanone, from the common intermediate **204**.

This key sequence involved the use of MacMillan’s catalyst III in the presence of alkynal **26** and diene **202** to achieve tetracyclic intermediate **204** in excellent enantioselectivity (97% *ee*) via elimination and subsequent stereoselective addition of the iminium complex **203**. Further transformations afforded (−)-strychnine by an eight-step sequence including decarbonylation reaction, isomerization, and an intramolecular Heck cyclization. The total synthesis of other five structurally complex alkaloids were accomplished via an organocatalytic cascade strategy. A similar strategy to build the enamine moiety was employed in 2009 by the same group [135] in their elegant total synthesis of (+)-minfiensin with excellent enantioselectivity (96% *ee*). It is especially impressive that the naphthyl-substituted MacMillan’s catalyst III was found to be significantly superior with respect to enantioselectivity compared to the corresponding phenyl-substituted MacMillan’s catalyst II. It is interesting to note that the key organocatalytic cascade strategy not only provided the collective synthesis of highly complex architectures but also indicated the unprecedented synthetic efficiency (the shortest asymmetric synthesis of strychnine).

**Scheme 51:**
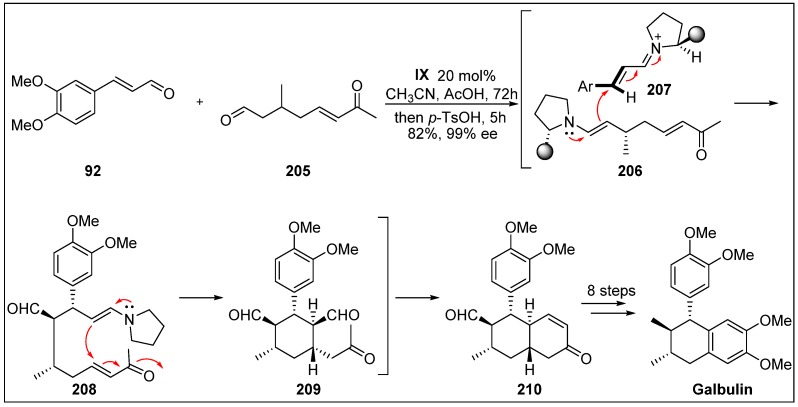
Total synthesis of (+)-galbulin.

In 2012, the Hong group [136] accomplished the first enantioselective total synthesis of tetrahydronaphthalene lignan (+)-galbulin (Scheme 51), which was first isolated in 1954 from *Himantandra baccata* as well as *Himantandra belgraveana* [137]. The key reaction in this elegant total synthesis was a highly enantioselective domino Michael addition/Michael addition/aldol condensation.

This key sequence employed Jørgensen’s catalyst IX in the presence of α,β-unsaturated aldehyde, ketoaldehyde, AcOH and *p*-TsOH to generate the hexahydronaphthalenone with superb enantioselectivity (99% *ee*) via intermolecular Michael addition of **92** and **205**, followed by intramolecular Michael addition of **208** and subsequent aldol condensation of **209**. Intermediate **210** was then successfully converted into (+)-galbulin in a series of high-efficiency transformations including selective allylic oxidation, epoxidation, and aromatization.

**Scheme 52:**
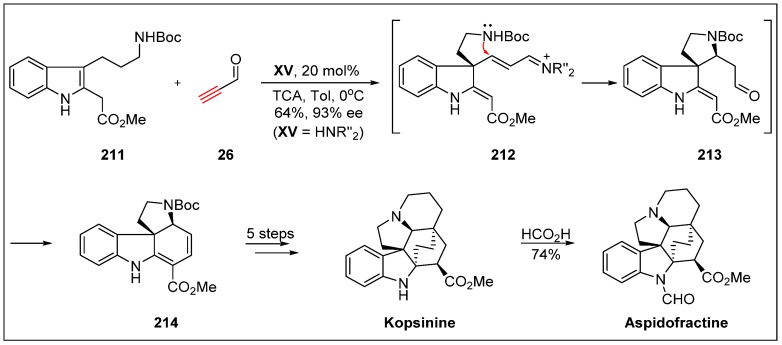
Total synthesis of (-)-kopsinine provided (-)-aspidofractine.

In 2014, the Wu group [138] also reported a highly valuable organocatalytic cascade strategy for the enantioselective synthesis of *Kopsia* family monoterpene indole alkaloid (−)-kopsinine [139], which containing a unique bicycle [2.2.2]octane architecture (Scheme 52). The key reaction in this elegant total synthesis was a highly enantioselective tandem intermolecular Friedel-Crafts/intramolecular *aza*-Michael addition/cyclization reaction using organocatalytic cascade strategy.

This key sequence required the use of Jørgensen’s catalyst XV in the presence of alkynal **26** and tryptamine **211** to achieve tetracyclic spiroindoline **214** with excellent enantioselectivity (93% *ee*) via diastereoselective *aza*-Michael of **212**, followed by cyclization of **213** and spontaneous dehydration. Further transformations afforded (−)-kopsinine by a five-step sequence including Michael addition, Diels-Alder reaction, stereoselective hydrogenation, and intramolecular Heck cyclization. Further, *N*-formylation of (−)-kopsinine provided (−)-aspidofractine. It is noteworthy that this alternative process to (−)-kopsinine also highlighted the unique capability of the key organocatalytic cascade strategy.

## 3. Conclusions

As a burgeoning powerful tool, iminium ion activation of α,β-unsaturated aldehydes has provided novel methods to facilitate access to the complex architectures of natural products and pharmaceuticals in high enantiomeric excess. The field of chiral secondary amine catalysts has been intensively investigated by many groups and is becoming one of the most vibrant areas of total synthesis in recent years.

It should be noted that the strategies and methods of total synthesis described within this review provide impressive examples due to the unprecedented synthetic efficiency (such as the shortest asymmetric total synthesis of frondosin B; industrial scale asymmetric total synthesis of MK-0974; organocascade and collective asymmetric total syntheses of strychnine, aspidospermidine, vincadifformine, akuammicine, kopsanone and kopsinine). On the other hand, significant challenges remain (such as the industrial scale application in asymmetric drug synthesis). It can be expected that some new breakthroughs (such as lower catalyst loading, higher catalyst activity, more universal catalyst, and more clearly mechanism) in this field will be forthcoming.
